# Identification of low abundance microbiome in clinical samples using whole genome sequencing

**DOI:** 10.1186/s13059-015-0821-z

**Published:** 2015-11-27

**Authors:** Chao Zhang, Kyle Cleveland, Felice Schnoll-Sussman, Bridget McClure, Michelle Bigg, Prashant Thakkar, Nikolaus Schultz, Manish A. Shah, Doron Betel

**Affiliations:** Institute for Computational Biomedicine, Weill Cornell Medicine, New York, NY 10021 USA; Department of Medicine, Division of Hematology and Medical Oncology, New York-Presbyterian Hospital/Weill Cornell Medicine, New York, NY 10021 USA; The Jay Monahan Center for Gastrointestinal Health, New York-Presbyterian Hospital/Weill Cornell Medicine, New York, NY 10021 USA; Center for Advanced Digestive Care, New York-Presbyterian Hospital/Weill Cornell Medicine, New York, NY 10021 USA; Kravis Center for Molecular Oncology, Memorial Sloan-Kettering Cancer Center, New York, NY 10065 USA; Department of Epidemiology and Biostatistics, Memorial Sloan-Kettering Cancer Center, New York, NY 10065 USA

## Abstract

Identifying the microbiome composition from primary tissues directly affords an opportunity to study the causative relationships between the host microbiome and disease. However, this is challenging due the low abundance of microbial DNA relative to the host. We present a systematic evaluation of microbiome profiling directly from endoscopic biopsies by whole genome sequencing. We compared our methods with other approaches on datasets with previously identified microbial composition. We applied this approach to identify the microbiome from 27 stomach biopsies, and validated the presence of *Helicobacter pylori* by quantitative PCR. Finally, we profiled the microbial composition in The Cancer Genome Atlas gastric adenocarcinoma cohort.

**Electronic supplementary material:**

The online version of this article (doi:10.1186/s13059-015-0821-z) contains supplementary material, which is available to authorized users.

## Background

The human microbiome is a critical constituent of normal human physiology as well as in the pathogenesis of disease [1, 2], including malignancy [3–6]. Conservative estimates are that greater than 15 % of all malignancies may be attributed to microbiota [7], though the mechanisms by which the human microbiome contribute to malignancy remain largely unknown. There are several hypotheses, including microbiota inducing oxidative stress, altering immunosurveillance, changing local metabolism, affecting stem cell dynamics, or producing mutagenic metabolites [8]. Gastric cancer is one of the most commonly diagnosed gastrointestinal malignancies worldwide. It is responsible for nearly one million new cases and over 700,000 deaths annually [9]. The fatality:case ratio is high, demonstrating that the majority of patients diagnosed with gastric cancer will die of their disease, despite advances in drug therapy [10]. Gastric cancer is divided into specific subtypes based on specific epidemiology and risk factors [11] and, more recently, molecular profiles [11–13]. *Helicobacter pylori* is an endemic bacterial pathogen that infects nearly half of the world’s population [14], and is a WHO class I carcinogen for the development of gastric cancer, specifically the non-cardia, non-diffuse subtype of gastric cancer with an approximately threefold increased risk of malignancy in chronically infected individuals. *H. pylori* infection is believed to result in changes in gastric mucosal physiology and the epithelial host immune system [15–18]. Although several studies have examined causative features of *H. pylori*-associated gastric cancer, including bacterial virulence factors (CagA and VacA) [19–21], and host genetic alterations [22], what determines the consequence of *H. pylori* infection (i.e., whether mild gastritis, more severe peptic ulcer disease, or even gastric cancer) remains unknown.

Recently, a comprehensive molecular analysis performed through The Cancer Genome Atlas (TCGA) identified four distinct gastric cancer subtypes — (1) Epstein-Barr virus (EBV)-positive, (2) microsatellite instability, (3) genomically stable, and (4) chromosomal instability — based on their characterization using six molecular platforms: array-based somatic copy number analysis, whole-exome sequencing, array-based DNA methylation profiling, messenger RNA sequencing, microRNA sequencing and reverse-phase protein array [13]. The notable finding that EBV-associated gastric cancer is molecularly distinct from other gastric cancer subtypes speaks to the importance of external pathogens in this disease. However, this study did not report on the microbial composition in these tumor samples and the annotation of *H. pylori* was incomplete. Data are emerging on the diversity of the bacterial population (over hundreds of phylotypes [23, 24]) that reside in the stomach [23] and its dynamic composition associated with different disease states [25–27]. The microbial community in the stomach is typically limited by the low pH of the gastric lumen, which selects for acid-resistant bacterial populations, and usually limits the colonization densities to less than 1000 colony-forming units/g [24]. Due to the rise in pH caused by *H. pylori* urease activity [28] as well as other factors, *H. pylori* infection may have important effects on the composition of the gastric microbiome. However, the effect of *H. pylori* on the gastric microbiome is not well studied due to inadequate numbers of tissue samples and low bacterial content. Current methodology for profiling the microbiome from fecal or environmental samples is not directly applicable for detecting the microbiome from the upper gastrointestinal tract (i.e., stomach), in part due to the high content of human DNA in the sample that confounds microbial identification. Here we report on a systematic study utilizing whole genome sequencing (WGS) data to identify the microbiome composition from small endoscopic biopsy samples. We validated this methodology by quantitative PCR (qPCR) as well as comparison with other tools using datasets from the Human Microbiome Project (HMP), HapMap, human blood serum samples, a cystic fibrosis study, and TCGA studies. We further analyzed 27 gastric biopsies collected from patients with active *H. pylori* infection and with and without a history of treated *H. pylori* infection and found significant differences in their microbial content. We also performed similar analysis on the TCGA gastric samples and found that 40 % of the analyzed samples have strong evidence for *H. pylori* infection. This report details the methodology, computational pipeline, and validation of this novel approach, which can now be applied over serial endoscopic biopsies to examine temporal changes in the microbiome over time.

## Results

### Identification of microbiome content in mucosal biopsies

Microbial profiling of human mucosal tissues, such as oral cavities, skin, gastrointestinal tract, and urogenital tract, are typically performed by sequencing variable regions of the 16S ribosomal RNA gene [29, 30]. A limitation of this approach is that species identification is dependent on the extent of evolutionary diversification in those variable 16S regions where other genomic regions may be more informative for such speciation. More recently, whole genome shotgun sequencing was used to profile viral families [31, 32], for metagenomic studies [33, 34], and for the HMP [35]. The use of 16S and whole genome approaches for profiling the microbiome in human gastric biopsy samples is limited, however, because stomach mucosal biopsies contain mostly human DNA and a low abundance of microbial DNA, in contrast to samples collected from mucosal surfaces that are highly enriched for bacteria.

**Fig. 1:**
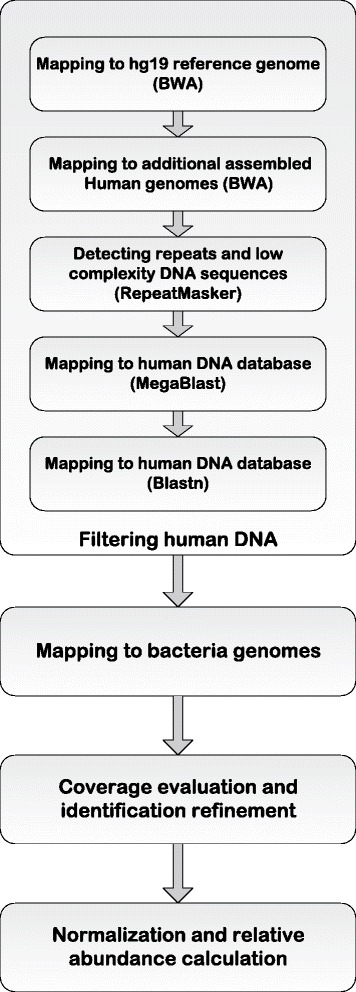
Workflow of the human DNA filtering and bacterial identification procedure. *BWA* Burrows-Wheeler Aligner

To overcome these limitations of microbiome identification from stomach biopsies and to maximize detection power, we performed WGS of 27 mucosal biopsy samples at roughly 10× coverage. On average, each library produced 400 million reads, of which 97 % were mapped to the standard human reference genome (Additional file 1). Initial profiling of the microbiome content from the remaining unmapped reads using clade-specific markers [36] resulted in a number of uncultivable bacteria species in roughly equal proportions in all samples (Fig. S1 in Additional file 2), such as *Candidatus Carsonella ruddii* (160-kbp genome,16.5 % GC content), *Candidatus Sulcia muelleri* (240-kbp genome, 22.5 % GC content), *Candidatus Zinderia* sp. (210-kbp genome, 13.5 % GC content) and *Wigglesworthia glossinidia* (700-kbp genome, 22.5 % GC content). These species are symbiotic bacteria that are restricted to insect hosts [37] with no previous report of human infection. Upon closer inspection we found that their genomes are short and have a low GC content, suggesting that their identification was incorrect, likely a result of short regions homologous with other bacteria or the human genome. We therefore devised a more stringent filtering approach adapted from the PathSeq pipeline [4]. Briefly, the pipeline is composed of successive filtering steps intended to remove the human-derived genomic reads (Fig. 1; Additional file 1). The reads are first mapped to the standard hg19 genome. The remaining unmapped reads are then mapped to a number of additional human assembled genomes and finally to a database of repeat regions. The remaining unmapped reads are then mapped to the National Center for Biotechnology Information (NCBI) full set of microbial reference genomes (see "Materials and methods").

**Fig. 2:**
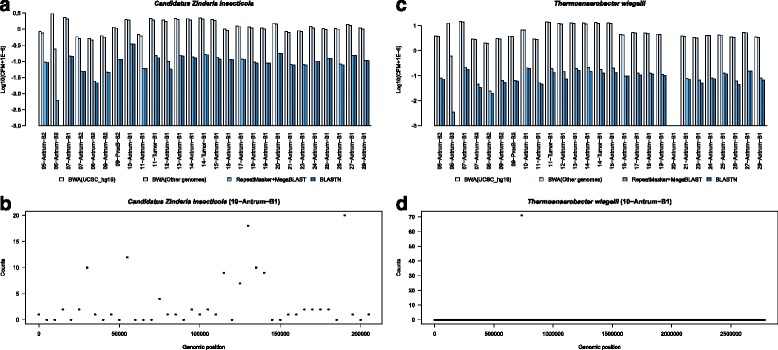
False bacteria identifications are readily identified by a drop in the number of mapped reads after filtering human sequences and inconsistent coverage. **a** Log-transformed counts per million (CPM) reads mapped to *Candidatus Zinderia insecticola* after each filtering step for each sample as outlined in Fig. 1. **b** Reads coverage map of *Candidatus Zinderia insecticola* in sample 10-Antrum-B1 after all filtering steps. Points represent the read counts in a 5-kb window. **c** Log-transformed CPM mapped to *Thermoanaerobacter wiegelii* after each filtering step for each sample. **d** Similar to (**b**), reads coverage map of *Thermoanaerobacter wiegelii* in sample 10-Antrum-B1. After all filtering steps, the coverage map indicates that reads map to only a single region of the genome. *BWA* Burrows-Wheeler Aligner

We used two specific mapping criteria for definitive identification of bacteria to reduce false detection. In the first, we evaluated the number of reads that map to the bacterial genome after the successive filtering steps. In many instances the number of reads that map to the bacterial genome drops sharply (often by more than 80 %) following the repeat masker and MegaBlast filtering (Fig. 2a, c). This removes the majority of reads with sequence similarities to repeat regions or human genomes. The second criterion is coverage uniformity of the bacterial genome. To that end we devised two measures of coverage variance that correct for sequencing depths and size of the genome (see "Materials and methods"). Both coverage measures were highly correlated.

**Fig. 3:**
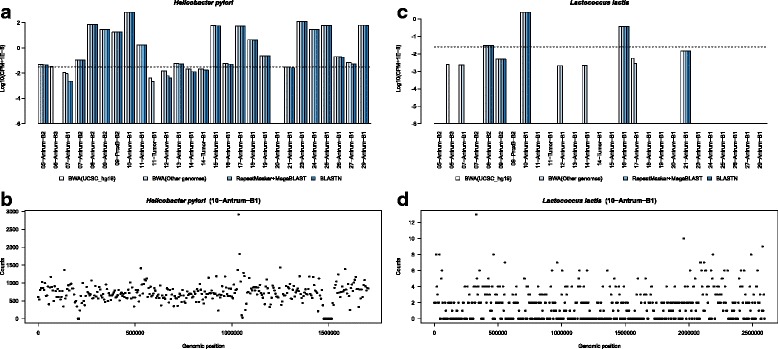
*H. pylori* (**a**, **b**) and *Lactococcus lactis* (**c**, **d**) retain consistent number of reads after each filtering step and the genomic coverage is uniform, indicating positive identifications. Similarly to Fig. 2, panels (**a**) and (**c**) indicate log-transformed counts per million (CPM) reads mapped to the respective bacteria and panels (**b**) and (**d**) contain read pileup along the genome in 5-kb segments. *Dashed lines* indicate minimum CPM values used in this study for positive identification of bacteria. *BWA* Burrows-Wheeler Aligner

These two filtering criteria were used to distinguish between falsely and correctly identified bacteria in the samples. For example, the number of reads mapping to *Candidatus Zinderia insecticola* and *Thermoanaerobacter wiegelii*, which were initially detected in high abundance in the antrum of patient 10, falls off dramatically with successive filtering (Fig. 2a, c), and the coverage of the mapped reads across each bacterial genome is not uniform (Fig. 2b, d), suggesting that these bacteria are not likely to be present in these samples. Alternatively, the data for *H. pylori and Lactococcus lactis* provide strong support for positive identification. These bacteria are not present in every sample, there is no dramatic reduction in number of mapped reads with successive filtering steps (Fig. 3a, c), and the coverage across each genome is more uniform (Fig. 3b, d).

### Microbial content validation by qPCR in biopsy samples

**Fig. 4:**
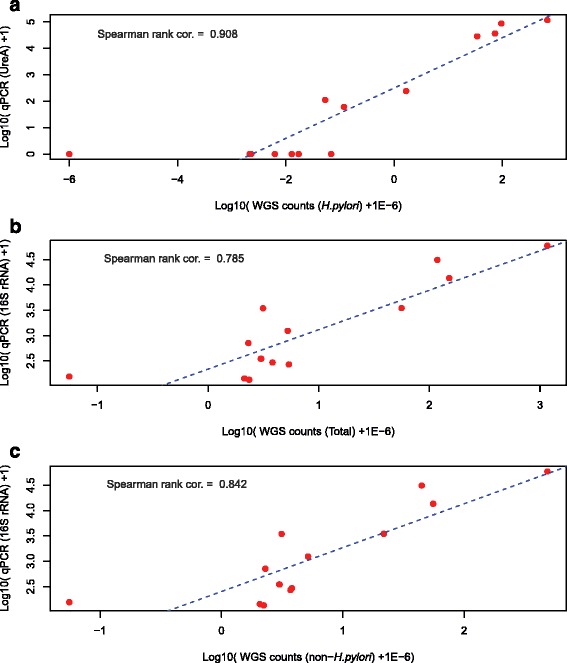
Correlation between qPCR-based bacterial quantification and WGS read counts. **a** Correlation plot between *H. pylori*-specific ureA gene-based qPCR results and WGS read counts (normalized by counts per million (CPM) reads) mapped to *H. pylori*. **b** Correlation plot between 16S rRNA gene-based qPCR results and total WGS read counts mapped to all bacterial genomes. **c** Correlation plot between 16S rRNA gene-based qPCR results and WGS read counts mapped to non-*H. pylori* genomes. In three cases qPCR measurements were derived from a sample that was adjacent to the WGS sample whereas in all other cases qPCR and WGS were performed on the same biopsy. Blue dashed lines are the linear regression models

To validate the results from WGS identification, we performed qPCR analysis to quantify the total bacteria content and *H. pylori* content by target amplification of a conserved region of 16S rDNA and the *H. pylori* specific ureA gene, respectively. We found excellent correlation between qPCR-amplified ureA gene and WGS *H. pylori* quantification (Fig. 4a; Spearman rank correlation 0.91), as well as high correlation with quantification of total bacteria using universal 16S primers and WGS (Fig. 4b; Spearman rank correlation 0.785, *p* value < 0.006). We note that although *H. pylori* accounts for the majority of the detected microbiome content in some samples, *H. pylori* read counts alone are poorly correlated to 16S qPCR-based total bacteria content, suggesting the presence of other (non-*H. pylori*) bacteria. This is further supported by the good agreement between the sequencing-based quantification and universal 16S qPCR when excluding *H. pylori* read counts (Fig. 4c; Spearman rank correlation 0.84).

### Comparison of microbiome detection methods in human samples

To further validate our microbial detection approach, we compared our methodology with MetaPhlAn [36] and Kraken [38], commonly used programs for microbiome detection, using samples from relatively sterile blood samples to evaluate false detection and bacteria-rich samples to evaluate concordance between the methods. We also included a number of tumor samples from various tissues to demonstrate that our approach is not restricted to tissue type or biased by bacteria content.

#### Comparison using sterile blood samples

We first selected three random samples (DRR000615, ERR055396, ERR047873) from the human HapMap project [39] to serve as negative controls. The HapMap project sequenced blood samples from donors across a wide range of human populations for the purpose of charting human genomic diversity. Erroneous bacteria identification is introduced from incomplete filtering of human reads or incorrect mapping of reads to bacterial genomes. As indicated by our initial analysis, some bacteria may be incorrectly detected because of similarities to human genomic loci. The HapMap samples serve as reasonable controls to assess false discovery since there should be little if any bacteria in blood samples.

Similar to the results from our gastric biopsy samples, we find that both Kraken and MetaPhlAn identified a number of bacteria species, such as *Candidatus Carsonella ruddii*, *Candidatus Sulcia muelleri*, *Candidatus Zinderia* sp. and *Wigglesworthia glossinidia*, that are likely misidentified due to contaminating human reads (Fig. S2 in Additional file 2). For example, in sample ERR055396, *Thermoanaerobacter wiegelii* and *Mycoplasma hyopneumoniae* had a large number of mapped reads while their genomic coverage was restricted to a few short regions (Fig. S3a, b in Additional file 2). In contrast, no detectible microbial species passed both the minimal number of reads and coverage uniformity criteria when using our own identification pipeline. *Propionibacterium acnes* was found in sample DRR000615; although 219 reads supported this identification, the genomic coverage is slightly below our cutoff for positive identification (Fig. S3c in Additional file 2). It is, however, consistent with previous reports that *P. acnes* is a common contaminant of blood cultures [40].

#### Comparison using bacteria-rich samples

To evaluate the performance of our method on bacteria-rich metagenomics samples, we profiled samples from the HMP [29] to serve as positive controls. The HMP is focused on mapping microbiome diversity across the human population at different body regions that are enriched for microbial species. We selected three posterior fornix samples from the HMP (SRS052620, SRS065347, SRS024428) with WGS data that were previously analyzed for microbiome content, and analyzed their reads using our computational detection procedure.

**Fig. 5:**
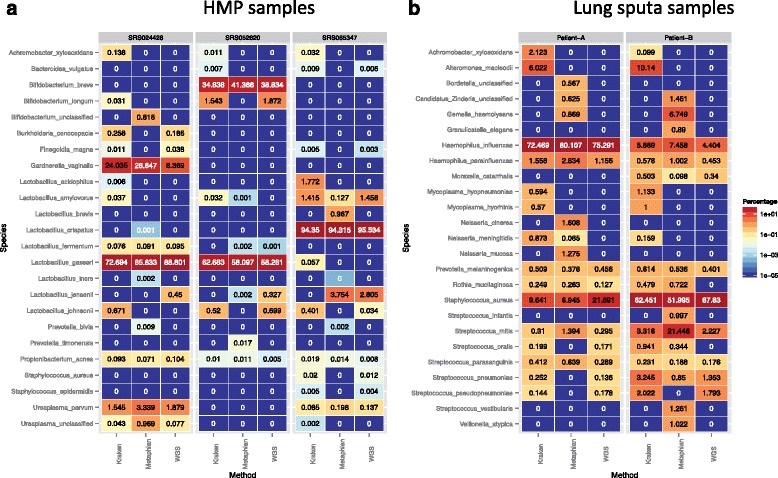
**a** Comparison of bacteria identified from three samples profiled by the HMP. **b** Comparison of bacteria identified from two lung sputa samples of cystic fibrosis patients. Both MetaPhlAn and Kraken and the procedure outlined in this study identify a consistent set of microbial species in the five samples (cosine similarity >0.95). Values represent percentage of bacteria reads found in each sample

Overall, the microbiome profile of the HMP samples generated by our analysis closely resembled the previously published microbial composition in both diversity and quantification (average cosine similarity 0.983, no statistical difference by t-test or Wilcoxon rank sum test; Fig. 5a; Fig. S4a, b in Additional file 2). The differences in species identification between the three methods are largely restricted to low abundance species. The sequence markers used by MetaPhlAn were derived from short unique bacteria regions that can miss low abundance species due to limited coverage. Kraken identifies bacteria species by matching k-mers to a database of bacteria k-mers (see "Comparison of microbiome detection methods" in Additional file 2). We identified several low abundance species that have not been reported by previous studies, and are likely true identifications as indicated by the uniform coverage of their genomes (Fig. S5 and "Comparison of microbiome detection methods" section in Additional file 2). Other previously identified species were not detected by our pipeline due to low read counts (<10) or insufficient genome coverage.

In addition to the HMP samples, we also analyzed two sputa samples from a published cystic fibrosis study [41]. Both of these samples contain a high percentage of bacterial content relative to biopsy samples, and serve as another positive control. The three methods reported very similar microbiome profiles (cosine similarity >0.92; Fig. 5b), where, similarly to the HMP samples, the discrepancies between the methods are attributed to differences in identification of low abundance species (see "Comparison of Microbiome Detection Methods" in Additional file 2). These results indicate that our pipeline has a similar capacity to detect microbiomes from the regular metagenomics samples as MetaPhlAn and Kraken.

#### Evaluation of human samples with low bacteria content to demonstrate clinical applicability

To test the applicability of our methodology for microbiome detection in clinical samples we performed additional evaluation on samples from a variety of different tissues collected in different clinical studies. We first analyzed WGS data from a serum sample collected in a non-malaria febrile illness study (SRR1106126) where *Haemophilus influenzae* was detected in one patient [42]. Consistent with the study report, both our pipeline and Kraken identified high levels of *H. influenzae* (82.9 % and 75.4 %, respectively) as well as *P. acnes* (17.1 % and 24.6 %, respectively), whereas no bacteria were identified by MetaPhlAn.

**Fig. 6:**
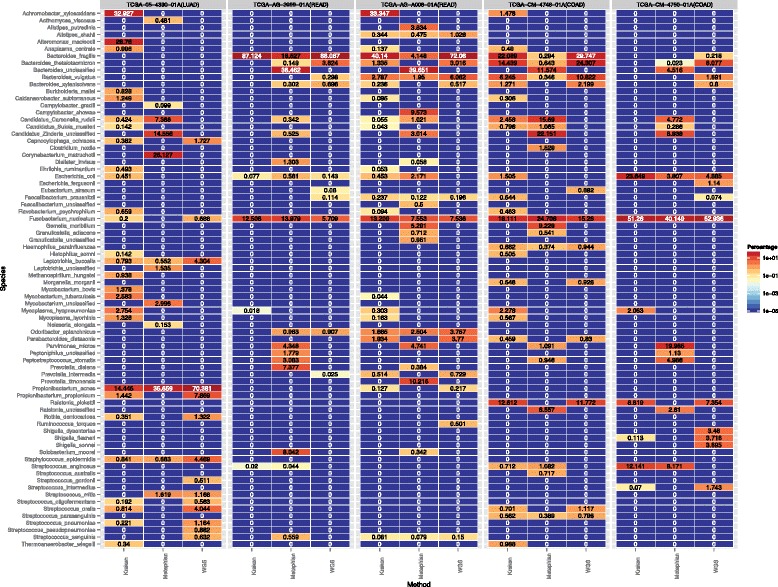
Comparison of identification by three methods from five TCGA tumor samples. Values represent percentage of bacteria reads found in each sample. *COAD* colon adenocarcinoma, *LUAD* lung adenocarcinoma, *READ* rectum adenocarcinoma

Next we analyzed WGS data collected from three TCGA studies (one from lung adenocarcinoma, two from rectum adenocarcinoma, and two from colon adenocarcinoma). Overall, all three methods identified the same dominant species from each sample, such as *P. acnes* in lung adenocarcinoma, *Bacteroides fragilis* in rectum adenocarcinoma, and *Fusobacterium nucleatum* in colon adenocarcinoma (Fig. 6; Fig. S6 in Additional file 2). Both MetaPhlAn and Kraken falsely identified bacteria that were also incorrectly reported in a previous evaluation, such as *Candidatus Carsonella ruddii*, *Candidatus Sulcia muelleri*, *Candidatus Zinderia* sp., *Thermoanaerobacter wiegelii* and *Achromobacter xylosoxidans*. Conversely, both methods did not identify species with significant genome coverage that were marked as a positive identification by our approach (Fig. S7 in Additional file 2).

To specifically address the impact of additional filtering of human DNA on all detection methods, we also performed comparison among the three methods on four gastric biopsy samples with full filtering of human reads. Even after extensive human DNA filtering steps, results from MetaPhlAn and Kraken included questionable identifications as measured by bacterial genome coverage. We conclude, therefore, that genome coverage evaluation is a key step to achieve accurate identification from these samples (Fig. S8 and " Comparison of Microbiome Detection Methods" section in Additional file 2).

Collectively, these results demonstrate that our methodology is able to identify microbial species in a wide variety of tissue types and that the main advantage of this approach over MetaPhlAn and Kraken is in samples with low levels of bacteria where the abundance of human DNA confounds bacteria detection.

### Clinical microbiome findings from gastric biopsy samples

Patients undergoing upper endoscopy who had no prior evidence of chronic inflammatory disease and no chronic use of nonsteroidal anti-inflammatory drugs were approached for enrollment and for research biopsies for microbiome analysis (Table 1). Twenty-two patients were enrolled, of which eight had active *H. pylori* infection as assessed by positive CloTest and confirmed on pathologic evaluation of the endoscopic tissue biopsy, and four of the eight actively infected patients also had prior history of *H. pylori* infection. The remaining 14 patients had no active *H. pylori* infection, although seven of them had prior history of infection. Our microbiome identification pipeline identified 18 patients with *H. pylori*, including all eight patients with active infection.

**Table 1:** Patient characteristics

Patient ID#	Gender	Age	*H. pylori* status	Prior H. pylori	*H. pylori* Pathology
05	F	49	Negative	Y (2009)	negative
07	F	50	Negative	Y (2010)	negative
08	M	50	Active Infection	Y	positive
09	M	36	Active Infection	Y (2003)	positive
10	F	69	Active Infection	Y (2009)	positive
11^a^	F	85	Negative	N	N/A (pathology did not test for h. pylori)
12	F	78	Negative	N	negative
13	M	57	Negative	N	negative
14^a^	M	36	Negative	Y (2013)	N/A (pathology did not test for h. pylori)
15	F	37	Active Infection	Y (2012)	positive
16^a^	M	63	Negative	N	N/A (pathology did not test for h. pylori)
17^a^	F	67	Negative	N	N/A (pathology did not test for h. pylori)
18^a^	M	71	Negative	N	N/A (pathology did not test for h. pylori)
19	F	46	Negative	Y (2009)	negative
20	M	56	Negative	Y (2004)	negative
21	F	43	Negative	Y	negative
23	F	51	Active Infection	N	positive
24	F	43	Active Infection	N	Unknown
25	F	26	Active Infection	N	positive
26	F	78	Negative	Y (2010)	negative
27	F	63	Negative	Y (2014)	negative
29	F	48	Active Infection	N	positive

^a^ Patient also with gastric cancer

**Fig. 7:**
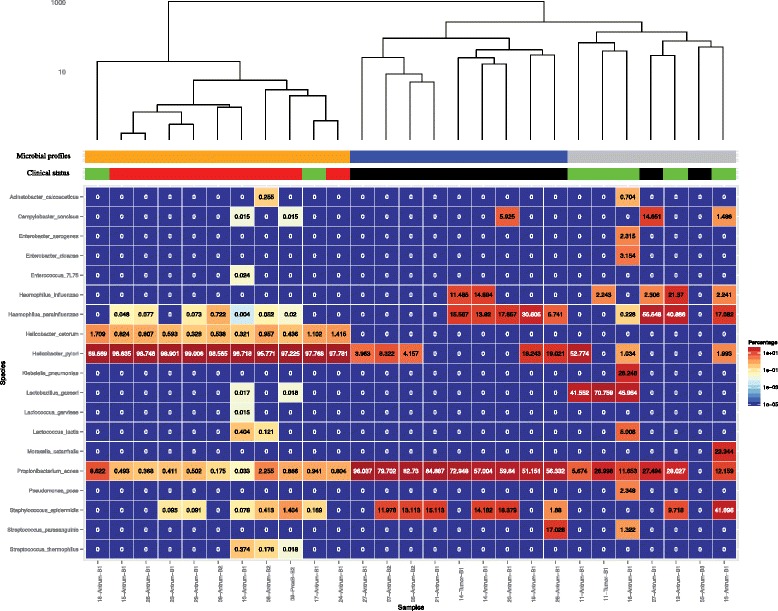
Bacterial relative abundance heatmap of profiled gastric biopsy samples. Hierarchical clustering of the 27 microbial profiles identified three primary clusters indicated by the *"Microbial profiles" bar*, with sample assignment to cluster p value <0.05 determined by 10 K bootstrap sampling. This grouping largely coincides with the clinical status of the samples: *red-labeled samples* were collected from patients with active *H. pylori* infection, *black-labeled samples* are from patients with prior history of treated *H. pylori*, and *green-labeled samples* are from patients with no history or present *H. pylori* infection

Hierarchical clustering of the 27 microbial profiles (from 22 patients) indicated three major groups (Fig. 7) that were largely consistent with the clinical annotation of the samples (Fig. 7).

The first group of samples (orange bar in Fig. 7) is characterized by a predominant signature of abundant levels of *H. pylori* (98.11 ± 0.96 %) that includes the nine samples from eight patients (IDs 08, 09, 10, 15, 23, 24, 25, and 29) with clinical validation of infection and two additional samples that had no prior clinical symptoms but contained similarly high levels of *H. pylori* (IDs 17 and 18).

The second group of samples (blue bar in Fig. 7) is characterized by a high proportion of *P. acnes*, as well as other less frequent species, including *Haemophilus parainfluenzae*, *H. influenzae*, and *Staphylococcus epidermidis. P. acnes* is the most dominant species in all nine samples and accounted for 51.2–96.0 % of relative content. Included in this group are five samples from five patients (IDs 05-Antrum-B2, 07-Antrum-B2, 19, 26, and 27) with intermediate levels of *H. pylori* that accounted for 4.2 %, 8.3 %, 18.2 %, 19.0 % and 4.0 % of their total bacteria content, respectively. These patients had previous history of *H. pylori* infection that was previously treated. None of these patients exhibited signs or symptoms of recurrent infection at the time of sampling. We also checked additional samples from two (IDs 05 and 07) of the above five patients with *H. pylori* read counts, but we did not detect *H. pylori* in additional adjacent biopsies (05-Antrum-B3, 07-Antrum-B1). Both of these adjacent biopsies had a low number of unmapped reads following the filtering of the human reads, although the starting number of sequenced reads was comparable to other samples, suggesting that these two biopsies had relatively low amounts of bacteria (Additional file 1). Although *H. pylori* was identified in only one biopsy collected from patient 07, another top bacterial species (*P. acnes*) was identified in both biopsies.

The third group of samples (grey bar in Fig. 7) is characterized by a broader bacterial diversity, in which there is no single bacteria that consistently dominates all samples, although three samples had a significant proportion of *Lactobacillus gasseri* and four had detectable *H. influenzae*. Two samples with previous infection history are included in this group (07-Antrum-B1, 05-Antrum-B3). The remaining four patients had neither history of infection nor clinical evidence for active infection, although three of them (ID 11-Antrum-B1, 13, and 16) had detectable levels of *H. pylori* bacteria in the gastric mucosa with 52.8 %, 2.0 % and 1.0 % *H. pylori* bacterial DNA, respectively. The presence of *H. pylori* in the above three cases may not represent active infection as their bacterial profile is distinctly different from the first group (Table 1).

Notably, we also had two gastric tumor biopsies (ID 11-Tumor-B1 and 14-Tumor-B1), neither of which contained *H. pylori* sequences. Patient ID 11 is of particular interest because the mucosal sample adjacent to the tumor had high levels of *H. pylori* that accounted for 52.8 % of mapped bacterial reads. The other top bacterial species in ID 11 (*Lactobacillus gasseri* and *P. acnes*) were identified in both tumor and mucosa, but *H. pylori* appears to have been excluded from the tumor.

Finally, *P. acnes* was identified in almost all biopsies at various levels. As a skin-enriched bacterium rather than a skin-specific bacterium, *P. acnes* could infect bones, joints, mouth, eye, brain, heart valves, and shunt [43]. *P. acnes* was also found in prostate samples and some studies suggest that inflammation caused by it contributes to prostate cancer [44, 45]. Other gastric microbiome studies also identified *P. acnes* in human stomach [46, 47]. In our evaluation of the detection pipeline we also identified *P. acnes* in samples from other studies, such as in blood serum [48] and lung adenocarcinoma [49]. Our biopsies were collected by a number of clinicians at different locations and dates, and sequenced at different dates. Therefore, contamination from a single source is not likely, although we cannot fully exclude this possibility. Because of the low number of reads mapped to *P. acnes* and the high similarity between *P. acnes* strains, we were not able to determine whether there is a single *P. acnes* strain common to all samples. Given the consistent low read counts of *P. acnes* across all samples, it might have been introduced to stomach mucosa through oral ingestion or oral contamination of the endoscope, rather than laboratory contamination.

### *H. pylori* and EBV infection status discovery in TCGA WGS data

We next analyzed the microbiome content in previously collected and sequenced TCGA gastric tumor samples, of which 37 of the 295 samples were profiled by low-pass WGS [13]. We began by examining our pipeline to identify EBV*-*associated gastric cancer as a validation of our pipeline. We found strong evidence for EBV infection in both samples that were previously classified as EBV-positive by TCGA genetic and expression profiling, providing further validation for our approach for microbiome detection (Table 2).

**Table 2:** Number of reads (in counts per million) mapped to *H. pylori* or EBV genomes in TCGA samples identified from WGS and RNA-seq data

	Tumor sample-WGS		Tumor sample-RNASeq		Tissue sample-WGS		Tissue sample-RNASeq	
Sample	*H. pylori*	EBV	*H. pylori*	EBV	*H. pylori*	EBV	*H. pylori*	EBV
TCGA-BR-4183	0.01	0.00	NA		0.01	0.00	NA	
TCGA-BR-4184	0.00	0.00	NA		0.01	0.00	NA	
TCGA-BR-4187	**0.76**	0.00	NA		**43.97**	0.00	NA	
TCGA-BR-4188	0.00	0.00	NA		0.00	0.00	NA	
TCGA-BR-4191	0.04	0.00	NA		**1.65**	0.01	NA	
TCGA-BR-4201	0.09	0.00	NA		**27.68**	0.03	NA	
TCGA-BR-4253	0.21	*6.36*	NA		**0.44**	0.00	NA	
TCGA-BR-4255	0.00	0.00	NA		0.04	0.00	NA	
TCGA-BR-4256	0.00	0.01	NA		0.00	0.00	NA	
TCGA-BR-4257	0.02	0.00	NA		**11.68**	0.00	NA	
TCGA-BR-4267	0.01	0.00	NA		**0.40**	0.00	NA	
TCGA-BR-4279	**6.99**	0.00	NA		**41.48**	0.00	NA	
TCGA-BR-4280	0.01	0.00	NA		0.01	0.00	NA	
TCGA-BR-4292	0.04	0.00	NA		0.00	0.00	NA	
TCGA-BR-4294	0.19	0.00	NA		**244.69**	0.00	NA	
TCGA-BR-4357	**0.34**	0.00	0.03	0.00	**64.43**	0.00	NA	
TCGA-BR-4363	0.00	0.00	**0.37**	0.00	0.00	0.00	NA	
TCGA-BR-4366	0.01	0.00	0.01	0.00	0.07	0.00	NA	
TCGA-BR-4367	0.01	0.00	0.03	0.00	0.01	0.00	NA	
TCGA-BR-4368	0.01	0.00	0.01	0.02	**16.97**	0.00	NA	
TCGA-BR-4369	0.00	0.00	0.00	0.17	0.03	0.00	NA	
TCGA-BR-4370	0.00	0.00	0.00	0.00	0.01	0.00	NA	
TCGA-BR-6453	**0.30**	0.00	0.01	0.00	0.19	0.00	0.03	0.00
TCGA-BR-6454	0.01	0.00	0.00	0.00	**17.95**	0.00	**2.72**	0.00
TCGA-CG-5720	**3.42**	0.00	**1.48**	0.00	**12.51**	0.00	**2.28**	0.00
TCGA-CG-5721	0.00	0.01	0.00	*0.43*	0.01	0.00	0.00	0.00
TCGA-CG-5722	0.13	*2.55*	0.04	*34.13*	**1.83**	0.00	0.00	0.22
TCGA-CG-5723	0.02	0.00	0.00	0.00	0.01	0.00	NA	
TCGA-CG-5724	0.03	0.00	0.00	0.00	**92.65**	0.00	NA	
TCGA-CG-5725	0.00	0.01	0.00	*0.61*	0.01	0.00	NA	
TCGA-CG-5726	0.01	0.00	0.00	0.00	0.00	0.00	NA	
TCGA-CG-5727	0.00	0.01	NA		0.01	0.00	NA	
TCGA-CG-5728	0.00	0.00	NA		**2.79**	0.00	**0.34**	0.00
TCGA-CG-5730	0.01	0.00	NA		0.00	0.00	0.00	0.00
TCGA-CG-5732	0.01	0.00	0.00	0.25	0.00	0.00	NA	
TCGA-CG-5733	0.01	0.00	NA		**2.01**	0.00	**0.63**	0.01
TCGA-CG-5734	**1.25**	0.01	0.03	0.17	0.00	0.00	0.00	0.00

Entries in bold are samples that are considered *H. pylori*-positive and entries in italics EBV-positive by a counts per million (CPM) reads threshold of 0.3. *NA* not applicable

Next, the tumor and adjacent tissue samples were examined for *H. pylori.* Since the *H. pylori* history of these samples was not available, we considered having at least 0.3 counts per million (CPM) reads mapped to *H. pylori* as evidence of *H. pylori* bacteria in the sample (with the same uniformity of coverage criteria as for our in-house samples; see "Materials and methods"). Strikingly, we found that 18 patients out of 37 (49 %) had significant evidence of *H. pylori* in either normal tissue or tumor. The overwhelming majority of *H. pylori*-positive samples were from the normal adjacent tissue. Only two patients had definitive *H. pylori* levels in both tumor and adjacent normal samples. Additionally, two other tumor samples (ID TCGA-BR-4187 and TCGA-BR-4357) had marginal support for *H. pylori* presence with 276 and 197 reads mapping to the *H. pylori* genome, respectively, whereas the adjacent normal tissue had an abundance of *H. pylori* (Table 2). We did identify one patient with *H. pylori* present in the tumor sample but not in the adjacent mucosa (ID TCGA-CG-5734). These microbial findings were further validated by analysis of the corresponding RNA-seq data (where available) for the presence of *H. pylori* and EBV transcripts with good agreement with WGS detection (Fisher’s exact test *p* value ≤ 2.9e-4; Table 2). The presence of *H. pylori* did not correlate with any clinical attributes of gender, tumor location, Lauren classification, or age, although the sample size is likely underpowered for detecting significant correlations (Fig. S9 in Additional file 2).

## Discussion

In this paper we present a systematic study of the unbiased and comprehensive identification of microbial species from small endoscopic biopsies by WGS. Accurate microbiome detection in these samples requires extensive processing to remove all possible reads that originate from human DNA. Careful comparison of microbiome detection with MetaPhlAn and Kraken using our own samples as well as samples from the HapMap project, the HMP, TCGA and other studies illustrates that all methods are confounded by high abundance of host DNA (Figs. S1–S9 and " Comparison of microbiome detection methods" in Additional file 2). Furthermore, because of the low coverage of bacterial genomes and sequence similarities between organisms, bacteria are often identified based on coverage along a narrow region of their genomes. To address this we included measures of uniform genomic coverage as additional evidence for bacteria identification.

In terms of microbiome profiling of biopsy samples, our results are highly consistent with qPCR quantification of *H. pylori* and universal 16S bacterial quantification. Using the same methodology, we were able to identify EBV-associated gastric cancer as was previously identified in the TCGA project. Finally, we also identified *H. pylori* in a significant portion of the mucosal samples collected as part of the TCGA project, and further identified the microbial species in the corresponding RNA-seq sample with highly significant correlation. Together, these data support our approach and demonstrate the feasibility of identifying the microbiome from small endoscopic tissue samples.

Notably, we identified *H. pylori* bacteria in the mucosa of patients who had been previously treated and were presumed cleared of this infection. It is conceivable that these are new infections; however, it is also possible that this identification represents persistent low-grade bacterial content of *H. pylori* in the gastric mucosa of previously infected and treated patients. The clearance rate of *H. pylori* by current standard practice of acid suppression and antibiotic therapy is 80–85 % [50, 51], although the rate of clearance may be declining due to the development of clarithromycin resistance [52, 53]. The presence of *H. pylori* in mucosa samples from patients with prior history of treated infections as well as in TCGA cohort raises the possibility that the tumorigenesis risk of *H. pylori* infection may be associated with the fact that the infection was not fully eradicated by conventional treatment, and that long-term chronic and asymptomatic infection may be the reason that current *H. pylori* treatment strategies to reduce gastric cancer risk have not proven successful [54]. The induced and long-term immune response facilitated by persistence of *H. pylori* may be a direct contributor to neoplastic transformation. Equally intriguing is the strong bias for *H. pylori* colonization in healthy mucosa tissues over tumor tissues seen in our sample and more strikingly in TCGA samples. Both observations are not statistically powered to draw broad conclusions but warrant follow-up studies.

In our survey, the gastric microbiome of patients with active *H. pylori* infection appears to be distinct from that in other samples. Specifically, the bacterial content of the gastric mucosa in actively infected individuals was significantly higher than the bacterial content of other individuals, and was dominated by *H. pylori* (98.11 ± 0.96 % of the bacterial reads). We did identify occult *H. pylori* in several additional samples, and note that it is not clear if these individuals will go on to develop a more profound active infection, or remain colonized. Besides being a disease-causing agent and a disease-protecting agent, *H. pylori* also has been considered as an indicator of changing human microbe ecology [55]. The high correlation between abundance of *H. pylori* and overall bacteria content (Fig. S10a in Additional file 2; Spearman rank correlation 0.861) may indicate that *H. pylori* infection may render the stomach mucosa a more favorable environment for bacterial colonization. It could be explained by *H. pylori* urease activity [28], and the consequent rise in gastric pH resulting in a more favorable environment for several bacteria, such as *S. epidermidis* (Fig. S10b in Additional file 2). Conversely, some bacteria were less abundant with increasing *H. pylori* content, such as *H. parainfluenzae* (Fig. S10c in Additional file 2) and *H. influenzae* (Fig. S10d in Additional file 2). Our validation studies suggest that WGS and the computational pipeline may detect bacteria below the limits of detection by qPCR, which may have implications in the future for defining *H. pylori* eradiation. Also of note is that we identified fewer phylotypes in our sample set (19 total phylotypes) than previously reported [23], but consistent with another report [56], and also consistent with the understanding of the relative germ-free environment of the stomach [57]. These differences are not likely related to the small patient sample set but rather differences in technique, including a lack of an amplification step in WGS. It may be possible to identify additional bacteria by WGS with higher sequencing coverage, but this is limited by lack of ability to validate, as we have approached our detection limit by qPCR with our current DNA sequencing coverage.

Previous studies have demonstrated extensive interaction between the gastric epithelium and the immune response to bacterial infection. For example, *H. pylori* infection activates proinflammatory cycloxgenase (COX) enzymes which regulate immune response and development [58]. Recent genomic studies have identified *Fusobacterium* in colorectal carcinoma [3], fusion of *Acinetobacter* DNA to human mitochondrial DNA in acute myeloid leukemia samples [5], and that EBV-associated gastric tumors are a genetically distinct class of gastric cancer [13]. Collectively, these studies suggest that bacteria and viruses may contribute significantly more than previously appreciated to the progression of various tumors.

## Conclusions

The microbiome, even when present in low abundance relative to human tissue, may have significant impact on human physiology.  However, it has been difficult to quantify and characterize the microbiome in clinical tissue samples where the bacterial content is low.  Here we present a new methodology to identify low abundant microbiome in small clinical tissue samples. The ability to directly characterize the microbiome from clinical biopsies opens a new possibility to investigate the interaction between microbial species and human tissues in a more direct way in a host of different tumor and tissue types, and longitudinally  across the management of a particular condition. 

## Materials and methods

### Gastric cancer and *H. pylori* research database

The Weill Cornell Medical College Gastric Cancer and *H. pylori* Research Database is a registry and tissue repository to examine the natural history of *H.* pylori infection in patients with and without gastric cancer. The primary aim of this research biobank is to provide an adequate tissue resource for the purpose of using modern molecular analytic tools to distinguish patients with chronic *H. pylori* infection who are at risk for subsequently developing gastric cancer from the vast majority of patients with *H. pylori* infection who do not develop malignancy. Subjects undergoing an upper endoscopy for clinical indications were approached for participation. All subjects provided written informed consent for participation in the Gastric cancer and *H. pylori* Research Database according to the Declaration of Helsinki prior to study enrollment. Samples were collected in accordance with the institutional ethical and clinical guidelines under institutional review board protocol 1203012274.

### Tissue collection and processing

Gastric mucosal biopsies from the antrum, proximal body and fundus were acquired for each enrolled patient and those with gastric carcinoma had additional biopsies taken at the tumor site as well as adjacent normal tissue. Biopsies were obtained using the Bard Precisor EXL coated disposable biopsy forceps (Bard International, Murray Hill, NJ, USA) and were immediately placed into individual sterile cryovials on dry ice and flash frozen while still in the endoscopic suite. The samples were then transferred to liquid nitrogen for prolonged storage. Genomic DNA was extracted from each gastric biopsy using an AllPrep micro kit (Qiagen, Hilden, Germany) according to the manufacturer's protocol. Samples were homogenized using a rotor stator homogenizer for less than 30 seconds. DNA concentration was measured for each sample using a Qubit**®** 2.0 Fluorometer (Life Technologies, Grand Island, NY, USA) and DNA quality was checked on a 1 % agarose gel stained with ethidium bromide. Samples were run alongside a 1-kb DNA Extension Ladder (Life Technologies, Grand Island, NY, USA).

### Whole genome sequencing

Extracted genomic DNA (1 μg) from each sample was given to the Weill Cornell Medical College Epigenomics Core for library preparation and subsequent WGS using an Illumina TruSeq DNA-seq DNA sample preparation kit and the Illumina HiSeq 2500 platform. Each sample was sequenced on a single flow cell lane as 50-bp paired-end reads. Homopolymers, adapters and distribution of base quality of raw sequences from each sample were investigated using FastQC (version 0.10.1). The filtered, non-human sequenced reads are available for download at BioProject ID PRJNA297869 [59].

### Quantitative PCR

Commercially available *H. pylori*-specific and universal bacteria16S qPCR assays were performed on patient samples according to the manufacturer's protocol and suggested cycling conditions (Primerdesign Ltd, UK). qPCR reactions were conducted in MicroAmp**®** 48-well optical plates (Life Technologies, Grand Island, NY, USA) in 25 μl volumes using 25 ng template genomic DNA, Perfecta**®** qPCR FastMix**®** II 10X master mix (Quanta Biosciences, Inc., Gaithersburg, MD, USA) and primers and probe supplied by Primerdesign Ltd with each assay, specific either for the ureA gene of *H. pylori* or the conserved region of the bacterial 16S gene. Samples were tested in triplicate along with negative and positive controls on the StepOne™ Real-Time PCR System (Life Technologies, Grand Island, NY, USA). Copy number was determined through extrapolation using the standard curve supplied with each kit.

### External datasets

HapMap samples (DRR000615, ERR055396, ERR047873), HMP WGS data (SRS052620, SRS065347, SRS024428), and a serum sample from a non-malaria febrile illness patient (SRR1106126) were collected from the NCBI Sequence Read Archive (http://www.ncbi.nlm.nih.gov/sra) and the HMP (http://www.hmpdacc.org/catalog/grid.php?dataset=metagenomic&hmp_isolation_body_site=urogenital_tract), respectively. WGS from a gastric adenocarcinoma study, a lung adenocarcinoma study, a rectum adenocarcinoma study, and a colon adenocarcinoma study and RNA-seq data from a gastric adenocarcinoma study were downloaded from the Cancer Genomics Hub (https://cghub.ucsc.edu/).

### Computational pipeline for microbial detection from WGS data

#### Filtering human DNA

This pipeline is based on the PathSeq [4] protocol for filtering human reads from WGS with a few modifications (Fig. 1). In the first step, the Burrows-Wheeler Aligner (BWA; version 0.6.2, with aln/sampe command) [26] was used to align the whole WGS data against the human reference genome (version hg19; http://genome.ucsc.edu/) using default parameters. In the second step, unmapped reads from the first step were aligned to three additional assembled human genomes available from the NCBI (hs_alt_CRA_TCAGchr7v2, hs_alt_HuRef, hs_ref_GRCh37.p5, build 37.3; ftp://ftp.ncbi.nlm.nih.gov/genomes/H_sapiens//Assembled_chromosomes/seq/, downloaded Feb. 06,2014 ) and to the Ensembl human genome reference (ftp://ftp.ensembl.org/pub/current_fasta/homo_sapiens/dna/) using BWA. RepeatMasker (version open-4.0, http://www.repeatmasker.org/ with –qq option) was used in the third step to identify repeat regions and low complexity sequences, and then any reads with three or more masked nucleotides were discarded for the next step. A combined human sequence database was generated for the last two steps and includes the following three datasets: i) the Ensembl *Homo sapiens* cDNA database (ftp://ftp.ensembl.org/pub/current_fasta/homo_sapiens/cdna/), ii) the NCBI *Homo sapiens* RNA database (ftp://ftp.ncbi.nih.gov/genomes/H_sapiens/RNA/) and, iii) the NCBI BLAST human genome database (ftp://ftp.ncbi.nlm.nih.gov/blast/db/FASTA/). In the fourth step, we performed alignments on the above database using MegaBlast (version 2.2.27) [60] with e-value cutoff 10^−7^ and word size 16. BLASTN (version 2.2.27) [60] was used as the last filtering step with the following parameters: cutoff expected value 10^−7^, word size 7, nucleotide match reward 1, nucleotide mismatch reward −3, gap open cost 5, gap extension cost 2. The remaining reads after all five filtering steps were used as the input for bacteria identification. All data used in this pipeline were downloaded on 6th February 2014.

#### Mapping to bacterial genomes

We collected 2736 bacterial whole genomes from NCBI (ftp://ftp.ncbi.nlm.nih.gov/genomes/Bacteria/). Due to the extremely unbalanced number of available strains of different bacteria, we retained the longest strain as the representative for each bacterium, resulting in a 1421-genome database that was indexed and used for bacteria identification. Bowtie2 [61] was used as the aligner to map the read to each bacterial genome (parameters --local -D 20 -R 3 -N 1 -L 32 -i S,1,0.50). According to the mapping results, each read will be labeled as an unmapped read, a unique mapping read or a multiple mapping read. Unmapped reads are reads that cannot be aligned to any bacterial genomes, and multiple mapping reads are reads which align to two or more bacterial genomes. We marked uniquely mapped reads as those mapping to only one bacterium genome regardless of whether they mapped to multiple locations of the same genome. The count of total bacteria reads is the sum of the uniquely mapped reads (Additional file 1).

#### Genomic coverage

For any bacteria with more than ten unique mapped reads, we calculated the read counts for each 5-kbp window and then computed two coverage measures to normalize for differences in library sizes and for differences in genome size:

1 $$ Var1=\frac{1}{n}{\varSigma}_{i=1}^n{\left(\frac{C_i}{\varSigma_{j=1}^n{C}_j}-\frac{1}{n}\right)}^2 $$

where C_i_ is the read count in window i, and n is the total number of 5-kb windows. In Eq. 1, the raw counts of each 5-kbp window are normalized by the total number of read counts mapping to corresponding bacteria to account for differences in number of reads mapped to each bacteria.

2 $$ Var2=\frac{1}{n}{\varSigma}_{i=1}^n{\left(\frac{n{C}_i}{\varSigma_{j=1}^n{C}_j}-1\right)}^2 $$

The second variation measure corrects for differences in genome size that can range from 139 kbp (*Tremblaya princeps*) to 13 Mbp (*Sorangium cellulosum*).

In this study positive bacteria identification was determined by ≥0.025 CPM reads, var1 ≤ 2 and var2 ≤ 1e-05.

#### Calculating Bacteria Abundance

In order to improve the sensitivity of relative abundance estimation, we used all reads mapped to bacterial genomes with PathoScope (version 2.0) [62], which is based on a Bayesian statistical framework to assign multiply mapped reads to the most probable bacterial source genome. To calculate relative bacteria abundance within each sample, read counts were normalized to the size of the corresponding bacterial genome to account for variation in bacterial genome size. The relative abundance of each bacterium is then calculated based on these normalized values.

### Source code

Source code files are available online (https://github.com/zhangch/WGSpipeline).

### *H. pylori* and EBV detection from RNA-seq gastric TCGA samples

BWA (version 0.6.2) was used to align the WGS data against the human reference genome (version hg19) with default parameters, and STAR aligner was used to align the RNA-Seq data against the human whole transcriptome. Unmapped reads were extracted and used for *H. pylori* and EBV identification. The EBV genome was added to the 1421-genome database that was generated for the analysis of biopsy WGS samples and Bowtie2 was used as the aligner with the same parameters as used in microbial detection from WGS data. *H. pylori* and EBV counts from each were normalized as CPM.

## Additional files

Additional file 1:
**Table listing the read counts of the gastric biopsy samples after each filtering step.** (XLSX 12 kb) Additional file 2:
**Supplementary figures and details of the comparison of identification results from three different methods.** (PDF 663 kb)

## Abbreviations

BWA: Burrows-Wheeler Aligner
EBV: Epstein-Barr virus
HMP: Human Microbiome Project
NCBI: National Center for Biotechnology Information
qPCR: quantitative polymerase chain reaction
TCGA: The Cancer Genome Atlas
WGS: whole genome sequencing
