# Supplementary material for: Identification of low abundance microbiome in clinical samples using whole genome sequencing
Source: Genome Biol. 2015 Nov 27;16:265. doi: 10.1186/s13059-015-0821-z (PMC4661937; doi:10.1186/s13059-015-0821-z)
Supplement: Additional file 2: — Supplementary figures and details of the comparison of identification results from three different methods. (PDF 663 kb) [file 13059_2015_821_MOESM2_ESM.pdf]

Supplementary Material: Identification of Low Abundance Microbiome in Clinical Samples Using Whole Genome Sequencing

Chao Zhang[1,2], Kyle Cleveland[2], Felice Schnoll-Sussman[2,3], Bridget McClure[4], Michelle Bigg[3], Prashant Thakkar[2], Nikolaus Schultz[5,6], Manish A. Shah[2,4,*], and Doron Betel[1,2,*]

[1]Institute for Computational Biomedicine, Weill Cornell Medicine, New York
[2]Division of Hematology/Oncology, Department of Medicine, Weill Cornell Medicine, New York
[3]The Jay Monahan Center for Gastrointestinal Health, New York-Presbyterian Hospital/Weill Cornell Medicine, New York
[4]Center for Advanced Digestive Care, New York-Presbyterian Hospital/Weill Cornell Medicine, New York
[5]Kravis Center for Molecular Oncology, Memorial Sloan-Kettering Cancer Center, New York
[6]Department of Epidemiology and Biostatistics, Memorial Sloan-Kettering Cancer Center, New York
[*]Corresponding authors

November 20, 2015

List of Figures

1 Initial MetaPhlAn's identification results on biopsy samples 2
2 Identification comparison of three methods on 3 HapMap samples 3
3 Examples of sequencing coverage of 3 species from HapMap samples 4
4 Scatterplots of identification results between any two methods for Human DNA enriched samples 5
5 Genomic sequencing coverage of 3 species from HMP samples 6
6 Scatterplots of identification results between any two methods for five TCGA tumor samples 7
7 Genomic sequencing coverage of *Ruminococcus torques* from a TCGA sample 8
8 The impact of additional filtering of human reads on microbiome detection 9
9 Comparisons of clinical and histological characteristics between *H. pylori* positive and negative TCGA samples 10
10 Correlations between abundance of *H. pylori* and other bacteria contents in 27 gastric biopsy samples 11

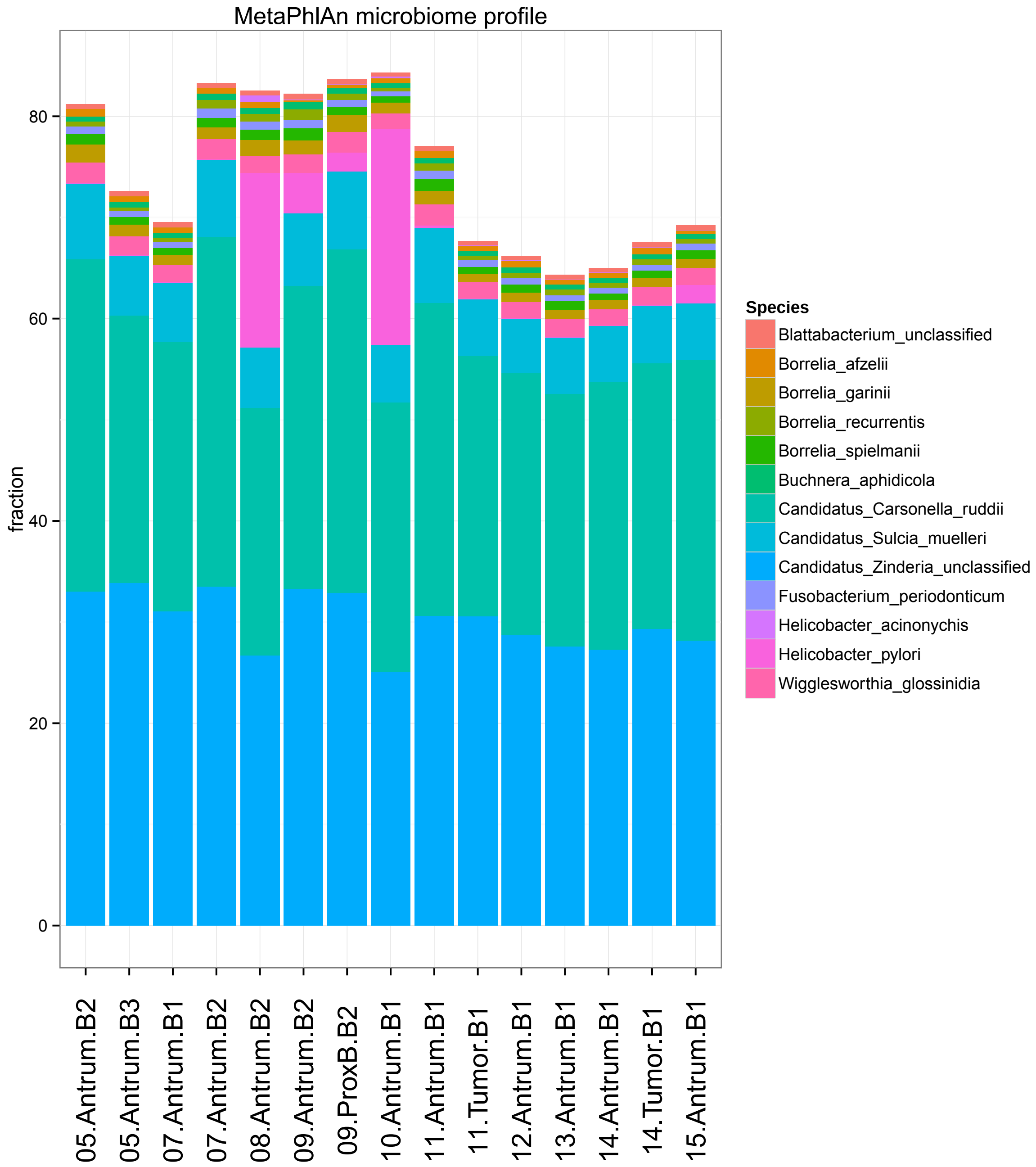

Figure 1: Initial microbiome detection identified a number of poorly characterized species that were subsequently determined to be incorrectly identified. Most of them could be removed by either additional filtering of human DNA or genome coverage evaluation (see Supplementary Figure 8 for results after stringent filtering of human DNA).

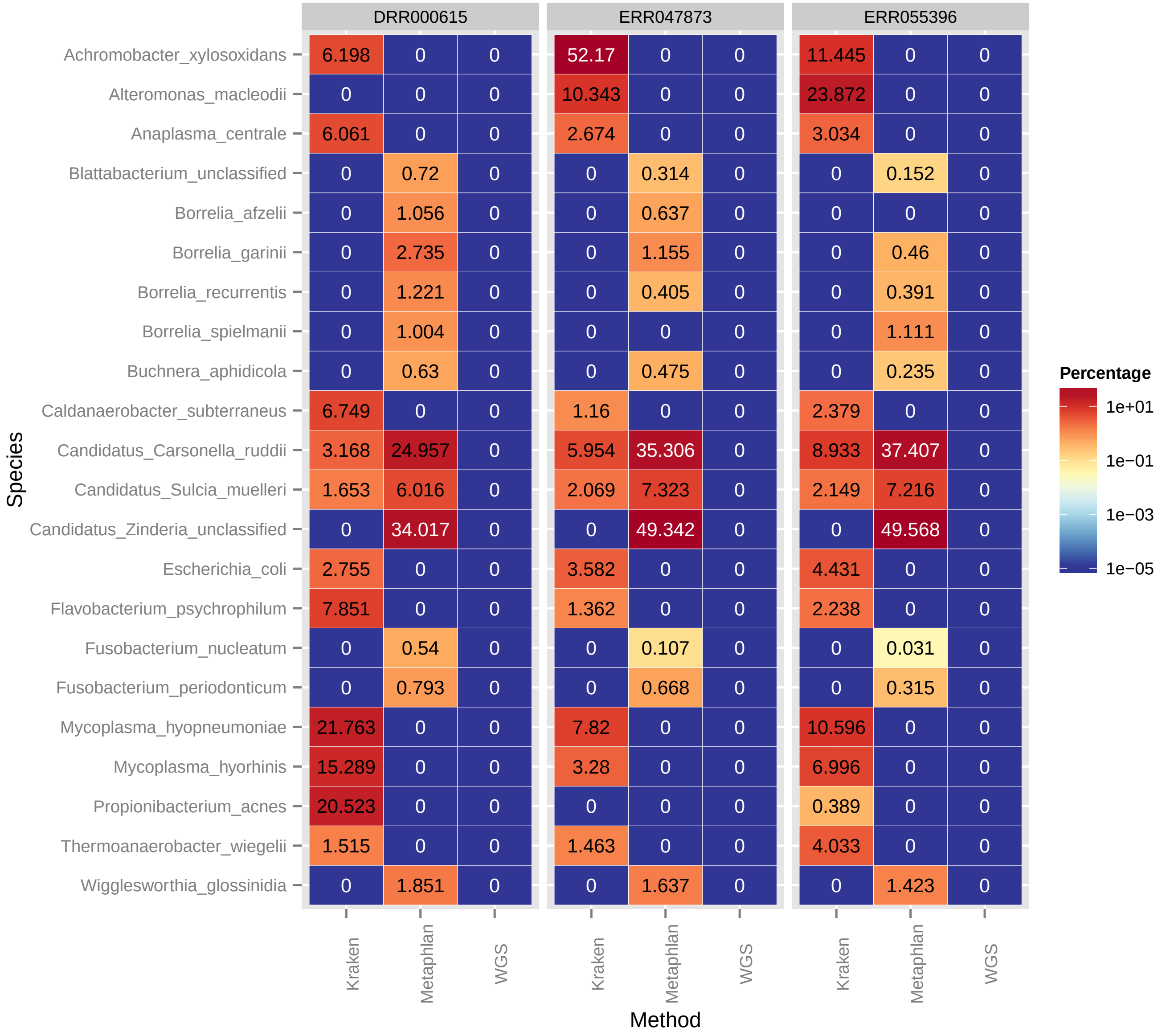

Figure 2: Comparison of microbiome detection using MetaPhlAn, Kraken and our pipeline (WGS) from 3 HapMap samples using full filtering of human reads (see Method section and Table 1 for similarity measures).

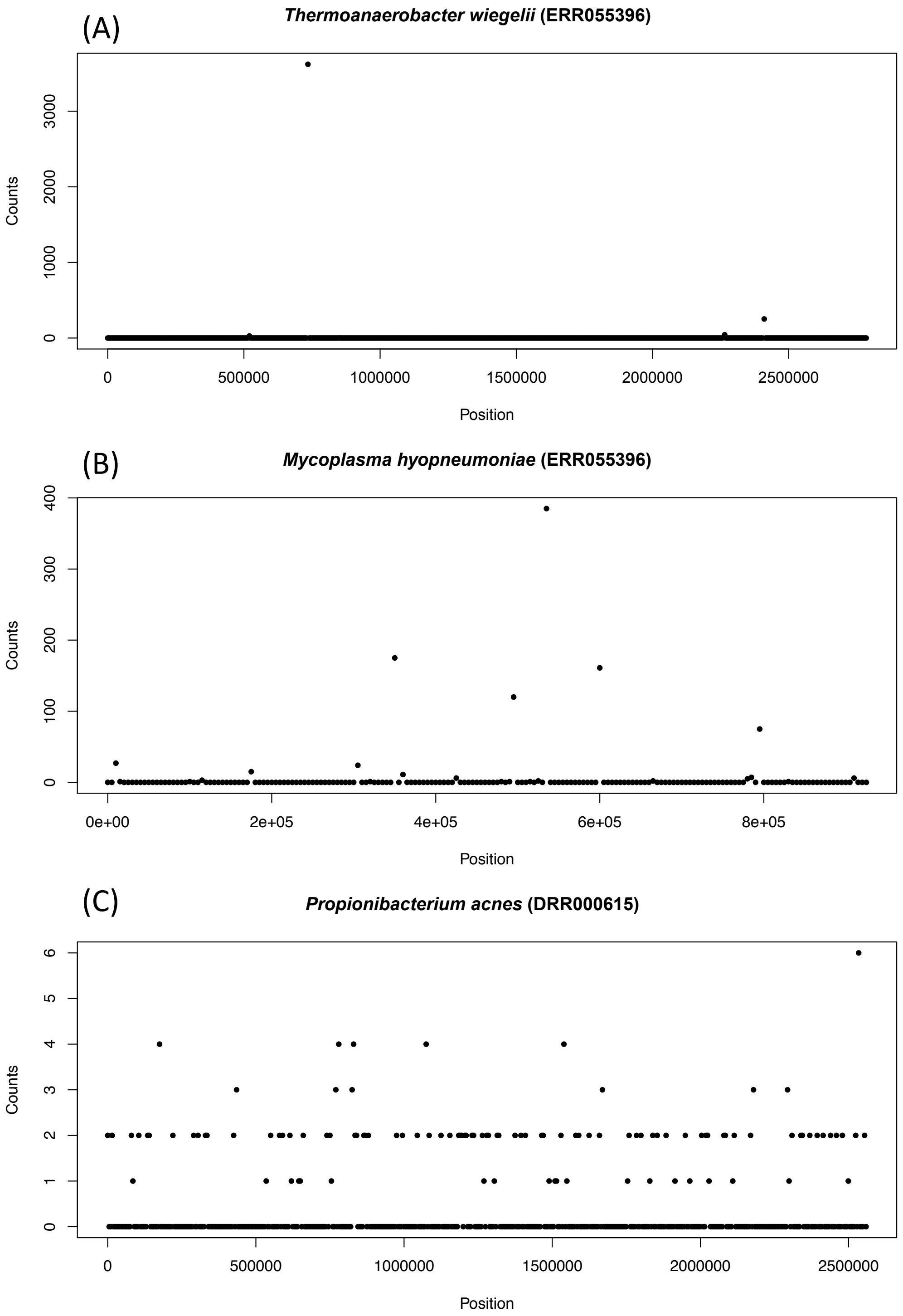

Figure 3: Reads coverage maps of *Thermoanaerobacter wiegelii* (A) and *Mycoplasma hyopneumoniae* (B) from HapMap sample ERR055396. Coverage is restricted to few narrow regions of the genome whereas the majority of the genome is not represented in the sequenced sample. *Thermoanaerobacter wiegelii* has limited sequence similarity to other bacterial organism and the two covered regions represent only a fraction of the unique genome. (C) Genomic sequencing coverage of *Propionibacterium acnes* from HapMap sample DRR000615 is more uniform throughout the genome and therefore more likely to be a correct identification.

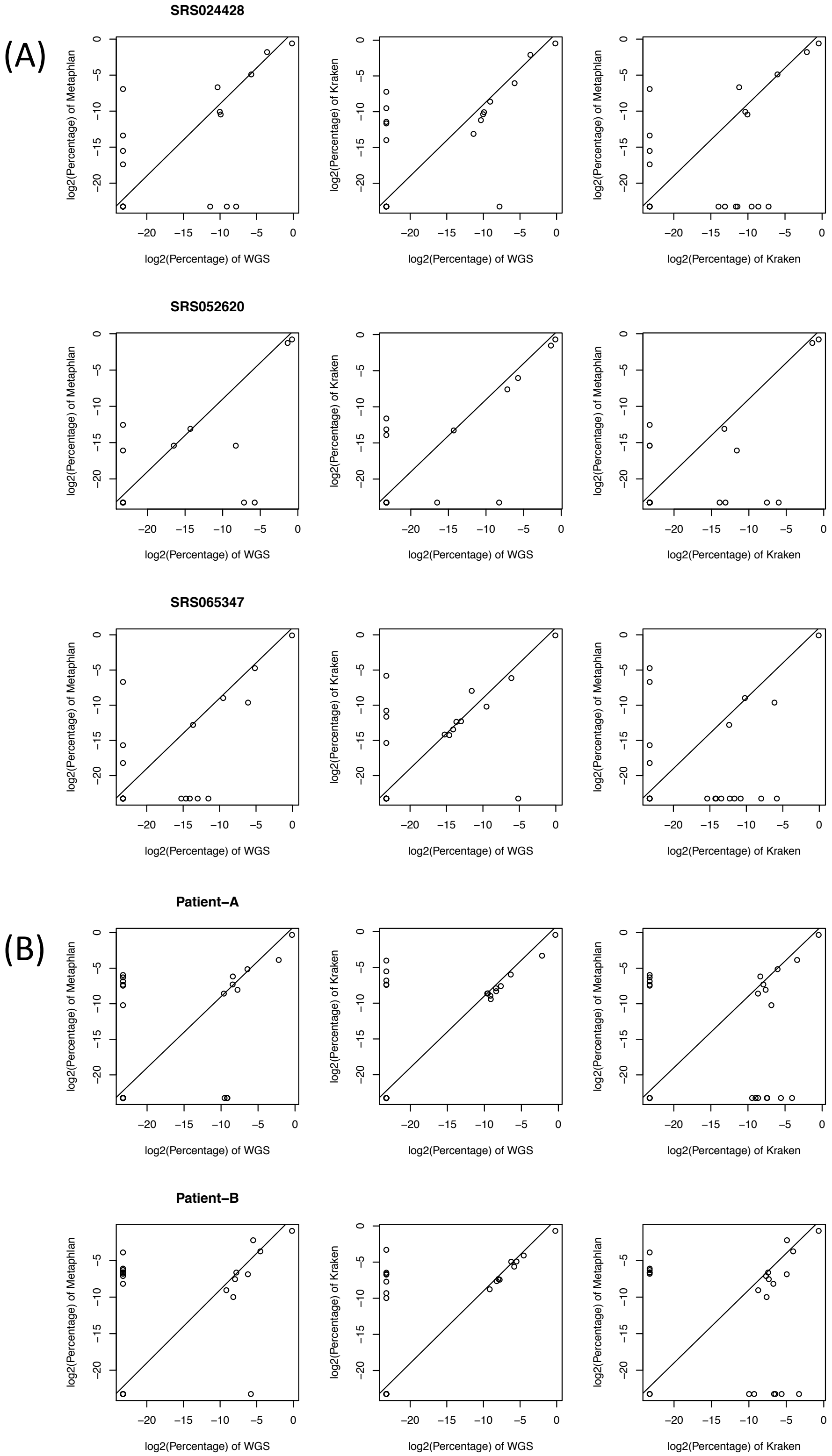

Figure 4: Scatterplots of identification results between any two methods: (A)Three HMP samples (B) Two sputa samples from a published Cystic Fibrosis study. The three methods reported very similar microbiome profiles.

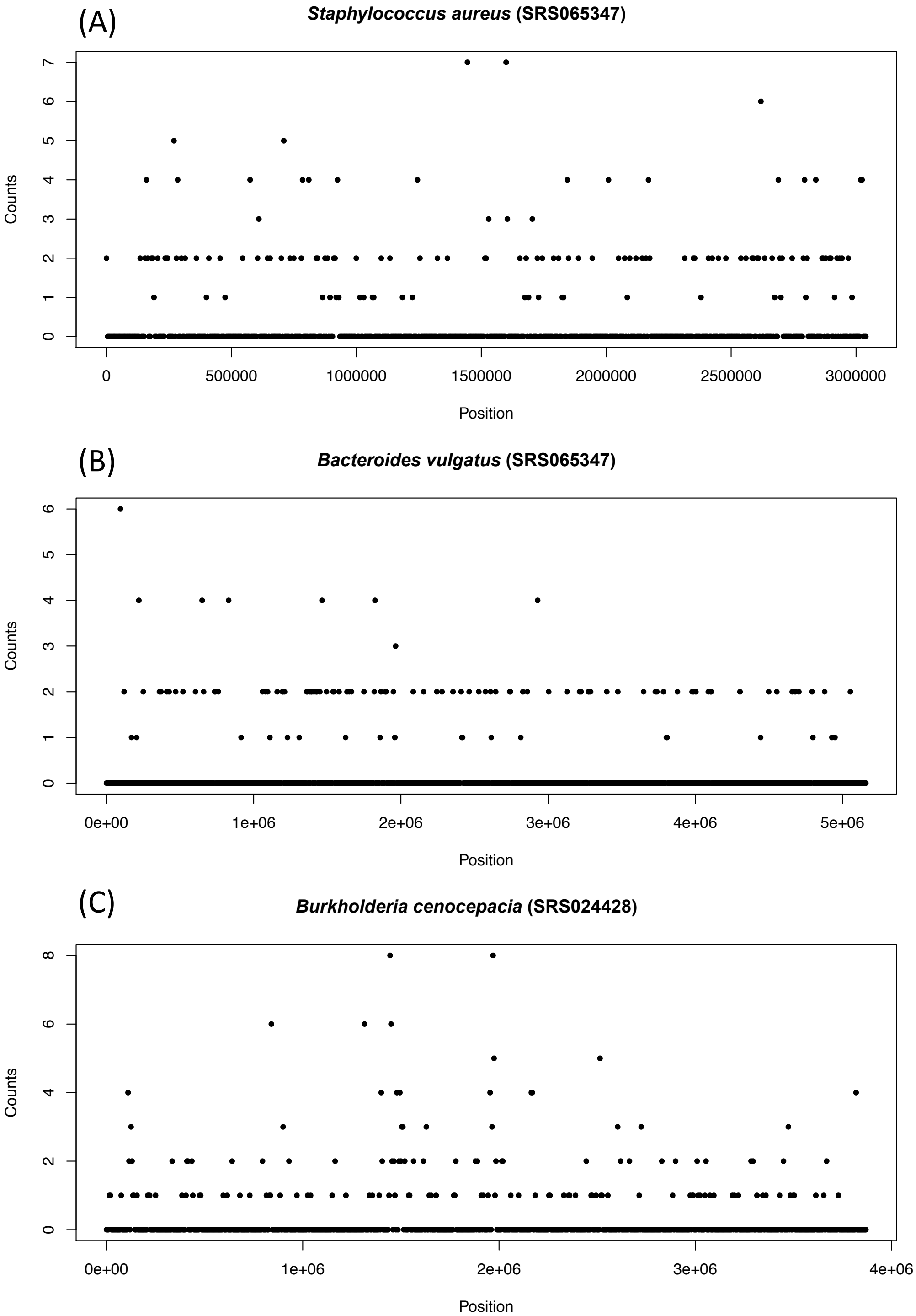

Figure 5: (A) Genomic sequencing coverage of *Staphylococcus aureus* from HMP sample SRS065347. (B) Genomic sequencing coverage of *Bacteroides vulgatus* from HMP sample SRS065347. (C) Genomic sequencing coverage of *Burkholderia cenocepacia* from HMP sample SRR02428.

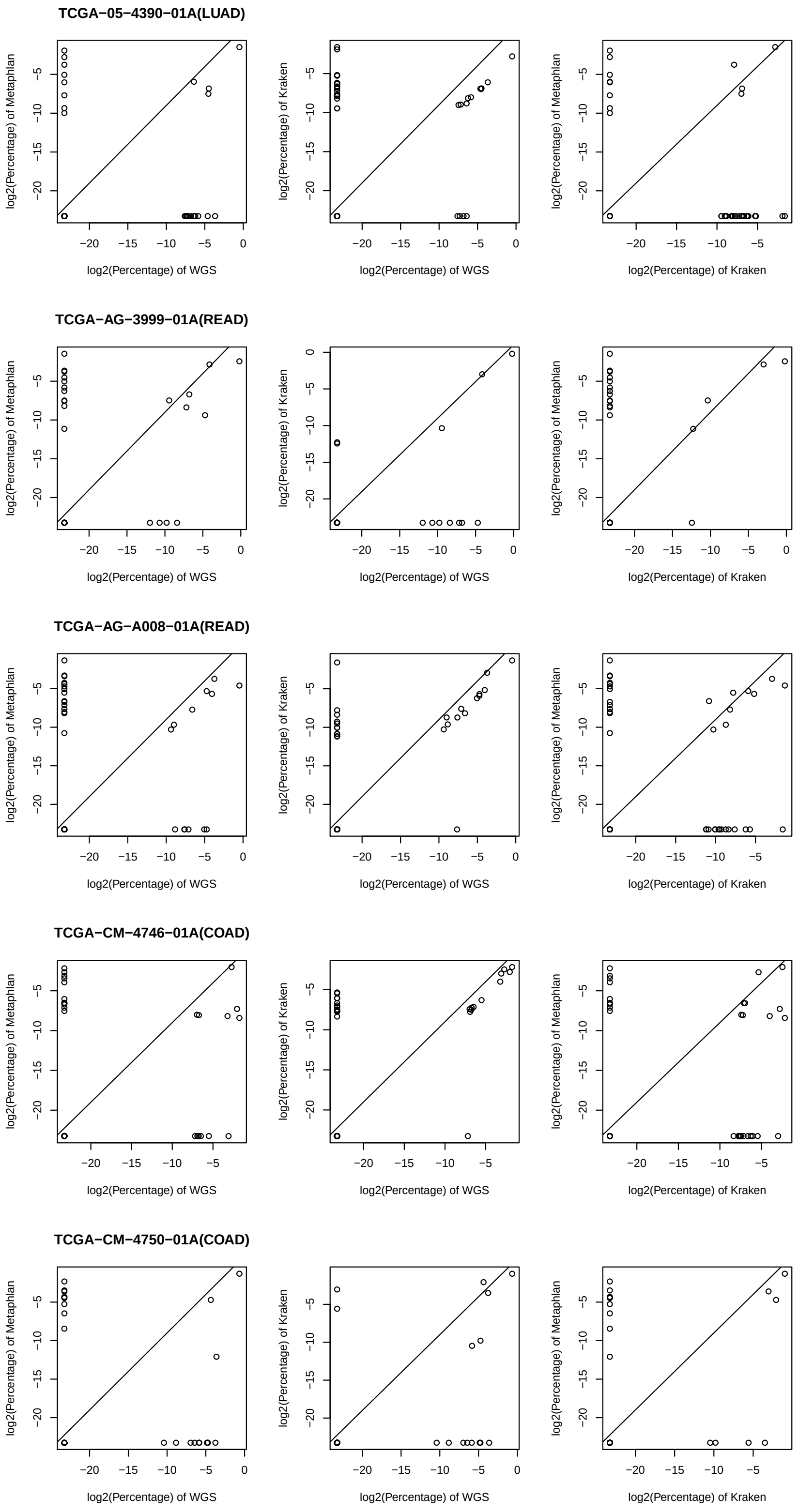

Figure 6: Scatterplots of identification results between any two methods for five TCGA tumor samples. Dominant species from each sample can be correctly detected by all three methods.

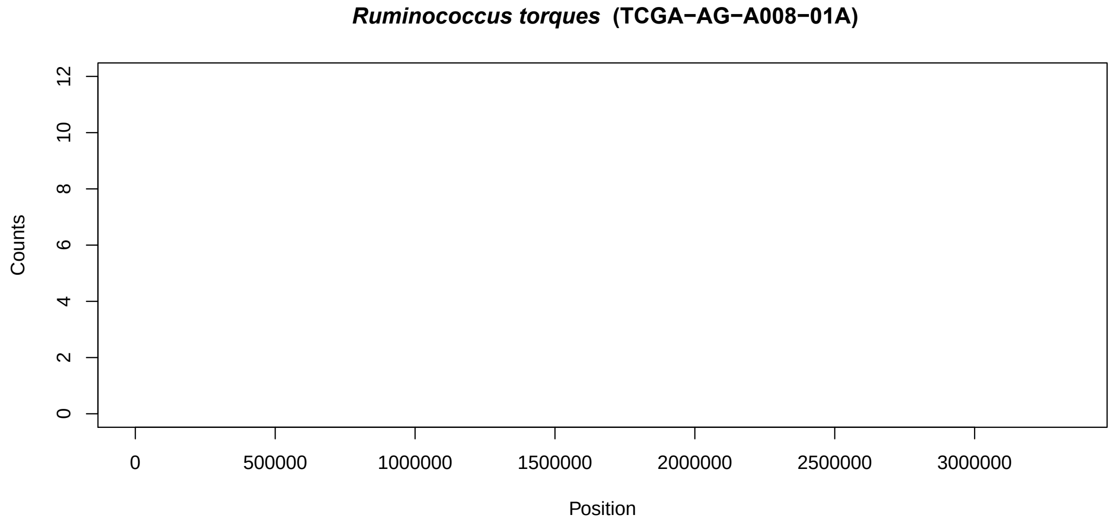

Figure 7: Genomic sequencing coverage of *Ruminococcus torques* from a rectum adenocarcinoma TCGA sample.

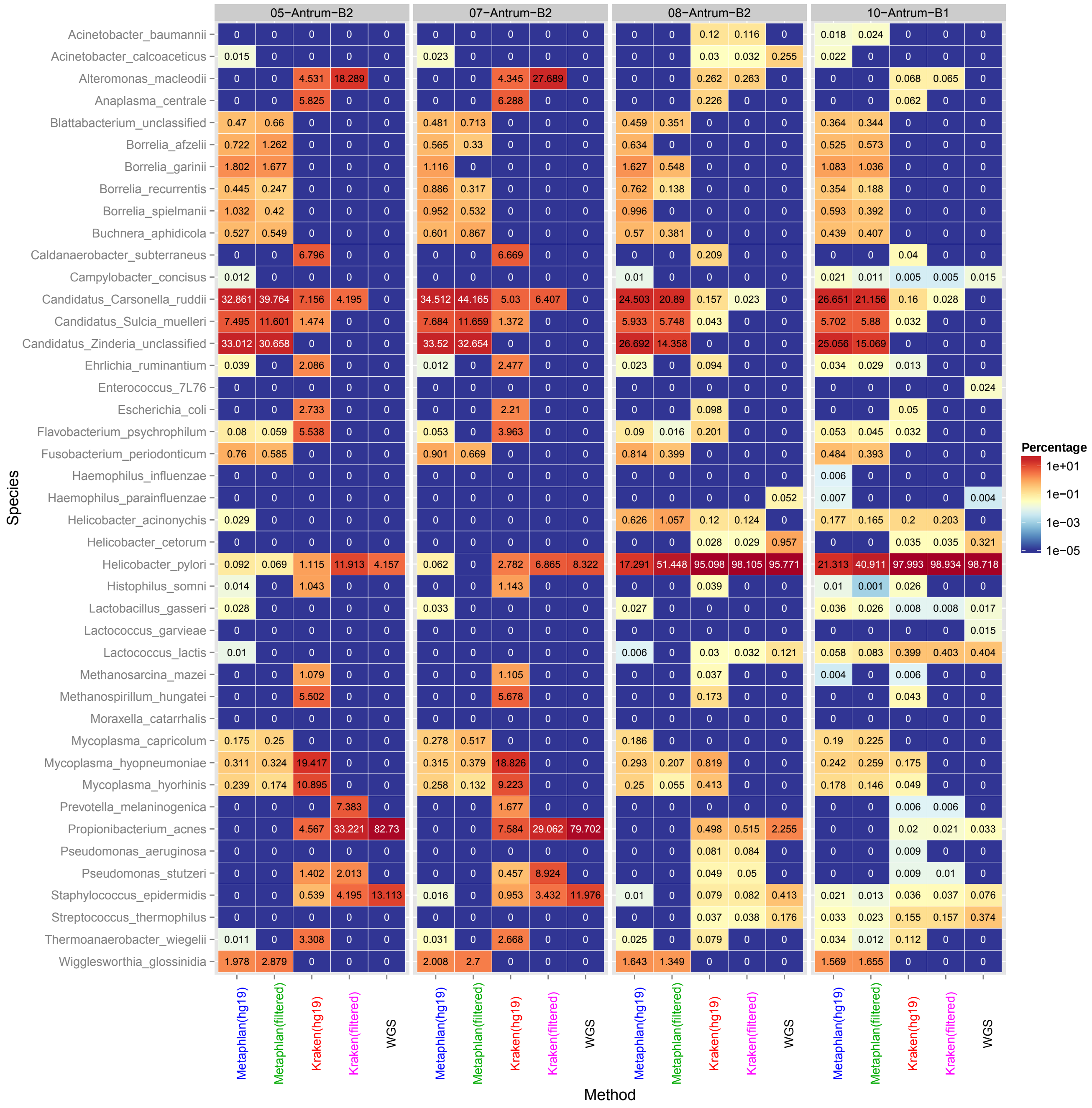

Figure 8: Evaluation of microbiome detection with additional filtering of human reads on four gastric samples. "hg19" columns represent the initial microbiome detection in four gastric biopsy samples by MetaPhlAn and Kraken after removing reads mapped to hg19. "filtered" are the identification results after more extensive filtering of human reads using additional human assemblies and repeat regions. Most top-ranked species were subsequently determined to be incorrectly identified and were removed by either filtering human DNA steps or genome coverage evaluation steps.

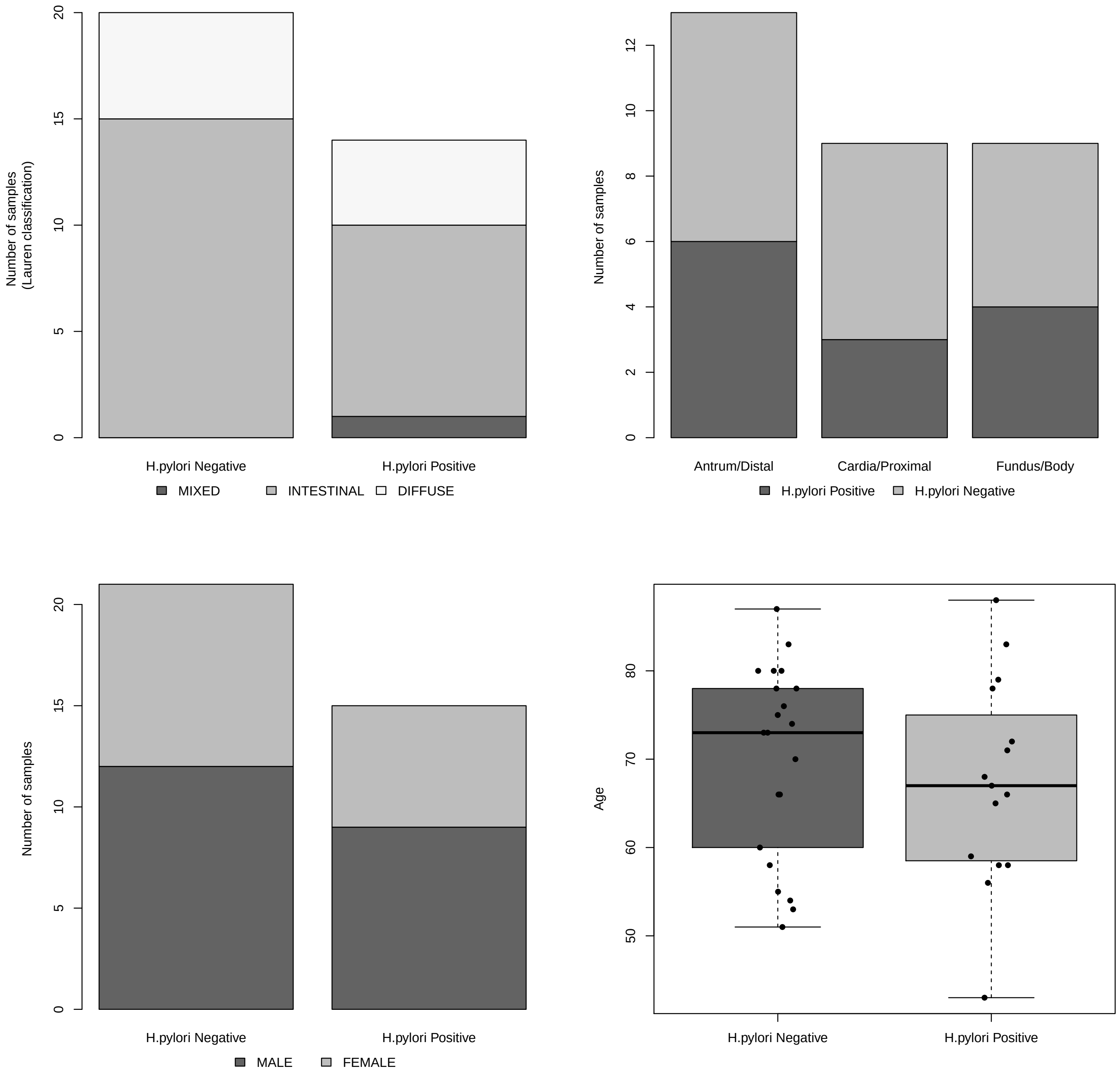

Figure 9: Comparisons of clinical and histological characteristics between *H. pylori* positive and negative TCGA samples did not identify statistically significant differences between the two patient groups.

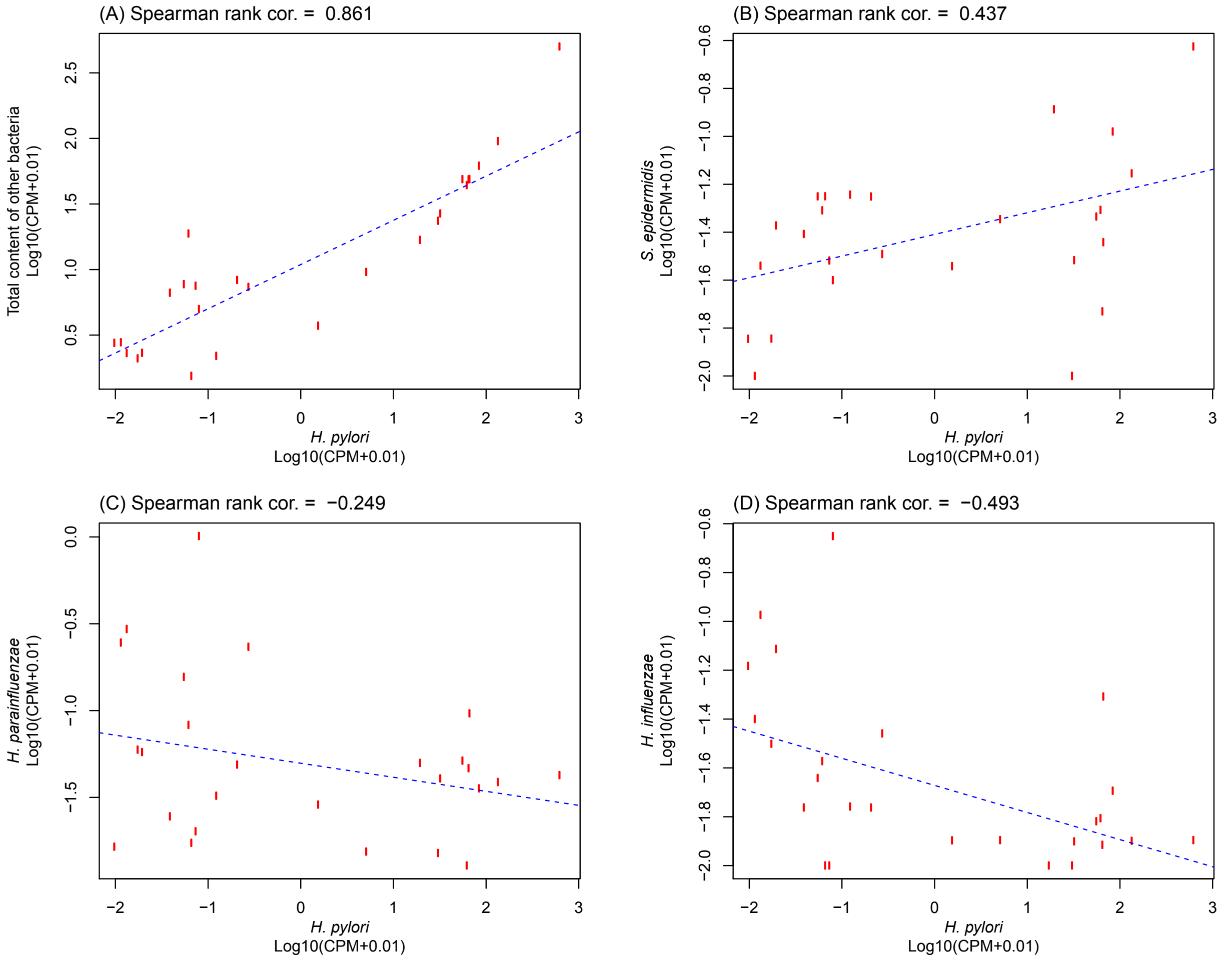

Figure 10: Correlations between abundance of *H. pylori* and other bacteria contents in 27 gastric biopsy samples.

Comparison of Microbiome Detection Methods

Detection of microbiome composition from gastric biopsies is confounded by the high abundance of host human DNA that can lead to misidentification of bacteria species due to similarities between human and bacterial segments as well as incomplete coverage of bacteria genome. We modified the PathSeq pipeline [1], which includes extensive human DNA filtering steps, with additional genomic coverage evaluation step to obtain accurate microbial identification from sample with low abundance of bacteria. These steps are particularly important in cases where host DNA is the most abundant species in the samples in contrast to other samples where bacterial DNA is predominant. In order to comprehensively evaluate the performance of our pipeline and illustrate the importance of these additional filtering steps in samples where bacterial cells are underrepresented relative to host cells, we compared our pipeline with two other widely used methods - MetaPhlAn [2] and Kraken [3] - using samples from gastric biopsies, the HapMap project [4], The Human Micriobiome Project (HMP) [5], The Cancer Genome Atlas (TCGA), a non-malaria febrile illness study, and a Cystic Fibrosis study.

All samples were aligned to the hg19 human reference genome with BWA (version 0.6.2), and the remaining unmapped reads were analyzed by our pipeline, MetaPhlAn (version 1.7.1, using default parameters and bowtie2db), and Kraken (version 0.10.5-beta, default parameters using MiniKraken DB).

Overall there is good concordance between the bacteria levels detected by our approach and those detected by MetPhlAn and Kraken as measured by cosine similarity (Table 1). The main differences are attributed to detection of low levels bacteria by MetaPhlAn and Kraken and the misidentification of remaining human reads.

Source	Sample	Similarity to MetaPhlAn	Similarity to Kraken
Biopsy (filtered with hg19)	05-Antrum-B2	9.65e-5	0.17
	07-Antrum-B2	1.77e-4	0.30
	08-Antrum-B2	0.42	0.99
	10-Antrum-B1	0.50	0.99
Biopsy (fully filtered human reads)	05-Antrum-B2	6.69e-5	0.83
	07-Antrum-B2	4.29e-7	0.70
	08-Antrum-B2	0.89	0.99
	10-Antrum-B1	0.84	1.00
HMP	SRS024428	0.95	0.97
	SRS052620	1.00	0.99
	SRS065347	1.00	1.00
Cystic Fibrosis (sputa samples)	Patient A	0.98	0.99
	Patient B	0.92	0.98
TCGA	TCGA-05-4390-01A (LUAD)	0.75	0.32
	TCGA-AG-3999-01A(READ)	0.43	0.99
	TCGA-AG-A008-01A(READ)	0.11	0.76
	TCGA-CM-4746-01A(COAD)	0.23	0.96
	TCGA-CM-4750-01A(COAD)	0.83	0.91

Table 1: Cosine similarity table comparing microbiome abundance detection between WGS and, MetPhlAn or Kraken. See Figure 5, Supplementary Figure 2, and Supplementary Figure 8 for abundance levels.

- Initail analysis on gastric biopsy samples

We initially applied MetaPhlan to 15 biopsy samples and the top 12 most abundant bacteria are listed in Supplementary Figure 1. Four species were determined to be incorrectly identified by genomic coverage evaluation (*Candidatus Carsonella ruddii*, *Candidatus Sulcia muelleri*, *Candidatus Zinderia* and *Wigglesworthia glossinidia*) and six additional species (*Borrelia afzelii*, *Borrelia garinii*, *Borrelia recurrentis*, *Borrelia spielmanii*, *Buchnera aphidicola* and *Fusobacterium periodonticum*) had insufficient coverage for positive identification.

Four of the samples (05-Antrum-B2, 07-Antrum-B2, 08-Antrum-B2 and 10-Antrum-B1) were evaluated by Kraken (Supplementary Figure 8) with similar results as MetaPhlan. All 13 top-ranked species were identified in all four samples and with the exception of *Helicobater pylori*, they were in similar abundance levels in all samples. With the exception of *H. pylori* levels, that were confirmed by clinical tests, there was little support for the presence of other bacteria in human samples by literature search or by careful examination of data.

- Evaluation of additional human DNA filtering steps on microbiome detection

  We speculated that many of the false bacteria identification are derived from human DNA with partial similarity to bacteria genomes. We therefore performed a more stringent filtering of human reads including mapping to additional assembled human genomes, all human reads in GenBank and repeat regions as outlined in the Method section. We then reevaluated MetaPhlan and Kraken against our approach using four gastric samples (Supplementary Figure 8).

  Overall, the number of reads mapping to the false identification falls off dramatically with successive filtering (Figure 2A,C), and the cosine similarity of each sample between our pipeline and other methods increased (Supplementary Table 1). However, a number of questionable identification are still reported by both MetaPhlan and Kraken due to low number of mapped reads or genomic coverage at few localized positions. Many of these can be excluded by simple coverage and uniformity filters. In addition, Kraken identification is based on k-mer matches to the target genome, which is sensitive to choice of k-mer length. For example, close inspection of 3 species (*Alteromonas macleodii*, *Prevotella melaninogenica*, and *Pseudomonas stutzeri*) identified by Kraken alone found that all of the reads had less than 70% similarity to their target genome (by BLAST and MEGABLAST searches) indicating that the parameters of k-mer matches may need to be modified for this specific identification task.

- Comparisons using HapMap samples

  HapMap [4] samples were used as negative control samples to evaluate the effect of human DNA on microbial identification. *Candidatus Carsonella ruddii* and *Candidatus Sulcia muelleri* were detected by both MetaPhlAn and Kraken as noted in the results section dor. Besides them, each method identified a unique set of bacteria. Careful examination of the genomic coverage of these species indicated non-uniform coverage suggesting that these bacteria are not likely to be present in the sample.

  MetPhlan identified: *Borrelia afzelii*, *Borrelia garinii*, *Borrelia recurrentis*, *Borrelia spielmanii*, *Buchnera aphidicola*, *Candidatus Zinderia*, *Fusobacterium nucleatum*, *Fusobacterium periodonticum*, *Wigglesworthia glossinidia* (Supplementary Figure 2).

  Similarly Kraken uniquely identified: *Achromobacter xylosoxidans*, *Alteromonas macleodii*, *Anaplasma centrale*, *Caldanaerobacter subterraneus*, *Escherichia coli*, *Flavobacterium psychrophilum*, *Mycoplasma hyopneumoniae*, *Mycoplasma hyorhinis*, *Thermoanaerobacter wiegelii* (Supplementary Figure 2).

Many of the bacteria species identified in the HapMap samples were identified in similar levels in the gastric samples suggesting that residual human reads may account for these identifications.

- Comparisons among three methods on HMP samples

  In order to evaluate the identification performance of our pipeline on normal metagenomics samples we randomly selected three posterior fornix samples from the HMP [5] as positive control samples. The identifications of our pipeline showed the very high cosine similarities to two other methods (Table 1).

  The majority of the discrepancy between our bacterial identification and those identified by MetaPhlAn and Kraken can be attributed to low read coverage or localized genomics coverage that were filtered out by our approach. There are two species that were were not identified by our approach (Figure 5). *Bifidobacterium unclassified* was identified by MetaPhlAn only in sample SRS024428 due to a genus-level marker that is present in MetaPhlAn database. *Achromobacter xylosoxidans* was identified in all three samples by Kraken and all of the reads mapped to *Achromobacter xylosoxidans* had less than 70% similarity to its genome (by BLAST and MEGABLAST searches) suggesting that it is a misidentification.

- Comparisons using sputa samples from Cystic Fibrosis patients

  Two sputa samples from a published Cystic Fibrosis study were used as the extra positive controls for evaluation. These two samples contain around 18% reads which cannot be mapped to hg19 reference genome. Although the relative abundance of microbial contents is lower than HMP samples, it is much higher than biopsy samples. The microbiome profile similarities detected by the three methods were highly consistent with each other, similarly to the HMP samples (Table 1).

  The majority of the differences between our pipeline and the two other methods were also limited to the low content species. Most of species not in our results didn't pass the genomic coverage criteria, such as, *Candidatus Zinderia*, *Neisseria mucosa*, *Neisseria meningitidis*, *Streptococcus infantis*, *Streptococcus vestibularis*, and *Veillonella atypical* from MetaPhlAn's results, *Alteromonas macleodii*, *Mycoplasma hyopneumoniae*, and *Mycoplasma hyorhinis* from Kraken's results. *Achromobacter xylosoxidans* from Kraken was identified as a false positive with the same reason in HMP samples. We missed three species *Gemella haemolysans*, *Granulicatella elegans*, and *Neisseria cinerea* detected by MetaPhlAn, since they are not included in our bacteria genome database.

- Comparisons using other human DNA enriched samples

  In order to estimate the applicability of microbial identification on other human DNA enriched samples which are similar to our gastric biopsy samples, we chose five tumor samples with high bacterial content from different TCGA studies. The results (Figure 6) revealed that most species with high abundance could be detected by all three methods. Some bacteria were missed by MetaPhlAn and Kraken, despite their uniform genomic coverage, that were previously reported as pathogen or commensal bacteria by previous studies. For example, Kraken did not identify *Bacteroides sp.* from TCGA-CM-4750-01A, and MetaPhlAn missed *Parabacteroides distasonis* (TCGA-AG-A008-01A) which is one of most common species isolated from human feces [6].

Although there is good agreement of the dominant species identified by all three methods, the cosine similarity between our pipeline and two other methods are very poor in several samples (Ta-

ble 1). This can be attributed to consistent misidentification by both MetaPhlAn and Kraken of few specific species. Kraken reported high abundance of *Achromobacter xylosoxidans* in many samples, and MetaPhlAn consistently identified *Candidatus Carsonella ruddii*, *Candidatus Sulcia muelleri*, and *Candidatus Zinderia*. These species were repeatedly detected by MetaPhlAn and Kreken in almost all samples evaluated in this study. However, their reported proportions in the low bacterial content samples are significantly higher than in other sample types.

Both MetaPhlAn and Kraken are optimized for fast identification of microbial species from microbial-enriched samples. MetaPhlAn maps sequenced reads to species specific genomic regions that have no similarity to other bacteria or human DNA. Kraken identifies bacteria by matching k-mers from sequenced reads to database of k-mer derived from bacterial genomes and assigning the reads to the lowest common ancestor. Both methods are significantly faster than our identification pipeline and preform well on HMP samples. However, in samples where bacteria is in low abundance sequence similarity among human and bacteria DNA, as well as low coverage of bacteria, can significantly impact species identification. The results from analysis of our biopsy samples as well as from HapMap, HMP, TCGA samples, and additional gastric biopsy samples illustrate that regardless of the species identification approach careful filtering of human reads and evaluation of genomic coverage are required for accurate identification of bacteria in samples with low bacteria levels.

References

[1] Kostic AD, Ojesina AI, Pedamallu CS, Jung J, Verhaak RGW, Getz G, Meyerson M: **PathSeq: software to identify or discover microbes by deep sequencing of human tissue**. *Nat Biotechnol* 2011, **29**(5):393–6.

[2] Segata N, Waldron L, Ballarini A, Narasimhan V, Jousson O, Huttenhower C: **Metagenomic microbial community profiling using unique clade-specific marker genes**. *Nat Methods* 2012, **9**(8):811–4.

[3] Wood DE, Salzberg SL: **Kraken: ultrafast metagenomic sequence classification using exact alignments.** *Genome biology* 2014, **15**(3):R46, [[http://www.ncbi.nlm.nih.gov/pubmed/24580807]].

[4] International HapMap 3 Consortium, Altshuler DM, Gibbs RA, Peltonen L, Altshuler DM, Gibbs RA, Peltonen L, Dermitzakis E, Schaffner SF, Yu F, Peltonen L, Dermitzakis E, Bonnen PE, Altshuler DM, Gibbs RA, de Bakker PIW, Deloukas P, Gabriel SB, Gwilliam R, Hunt S, Inouye M, Jia X, Palotie A, Parkin M, Whittaker P, Yu F, Chang K, Hawes A, Lewis LR, Ren Y, Wheeler D, Gibbs RA, Muzny DM, Barnes C, Darvishi K, Hurles M, Korn JM, Kristiansson K, Lee C, McCarrol SA, Nemesh J, Dermitzakis E, Keinan A, Montgomery SB, Pollack S, Price AL, Soranzo N, Bonnen PE, Gibbs RA, Gonzaga-Jauregui C, Keinan A, Price AL, Yu F, Anttila V, Brodeur W, Daly MJ, Leslie S, McVean G, Moutsianas L, Nguyen H, Schaffner SF, Zhang Q, Ghori MJR, McGinnis R, McLaren W, Pollack S, Price AL, Schaffner SF, Takeuchi F, Grossman SR, Shlyakhter I, Hostetter EB, Sabeti PC, Adebamowo CA, Foster MW, Gordon DR, Licinio J, Manca MC, Marshall PA, Matsuda I, Ngare D, Wang VO, Reddy D, Rotimi CN, Royal CD, Sharp RR, Zeng C, Brooks LD, McEwen JE: **Integrating common and rare genetic variation in diverse human populations**. *Nature* 2010, **467**(7311):52–8.

[5] Human Microbiome Project Consortium: **Structure, function and diversity of the healthy human microbiome**. *Nature* 2012, **486**(7402):207–14.

[6] Sakamoto M, Benno Y: **Reclassification of Bacteroides distasonis, Bacteroides goldsteinii and Bacteroides merdae as Parabacteroides distasonis gen. nov., comb. nov., Parabacteroides goldsteinii comb. nov. and Parabacteroides merdae comb. nov.** *International journal of systematic and evolutionary microbiology* 2006, **56**(7):1599–1605.
